# Challenges in Age-Related Macular Degeneration: From Risk Factors to Novel Diagnostics and Prevention Strategies

**DOI:** 10.3389/fmed.2022.887104

**Published:** 2022-06-06

**Authors:** Marco Lombardo, Sebastiano Serrao, Giuseppe Lombardo

**Affiliations:** ^1^Studio Italiano di Oftalmologia, Rome, Italy; ^2^Vision Engineering Italy srl, Rome, Italy; ^3^CNR-IPCF, Istituto per i Processi Chimico-Fisici, Messina, Italy

**Keywords:** age-related macular degeneration (AMD), lutein, adaptive optics, Resonance Raman (RR) spectroscopy, prevention

## Abstract

Age-related macular degeneration (AMD) is a chronic multifactorial eye disease representing the primary cause of vision loss in people aged 60 years and older. The etiopathogenesis of the disease remains uncertain, with several risk factors contributing to its onset and progression, such as genotype, aging, hypertension, smoking, overweight, and low dietary intake of carotenoids. Since the aging populations of the industrialized world are increasing rapidly, the impact of AMD in the socio-economical life-developed countries is expected to increase dramatically in the next years. In this context, the benefits of prevention and early disease detection for prompt and effective treatment can be enormous to reduce the social and economic burden of AMD. Nutritional and lifestyle changes, including dietary intake of xanthophyll pigments, such as lutein and zeaxanthin, no smoking, and regular exercise, are known to protect from risk of AMD progression from early to advanced disease stages. In this review, we present the clinical outcomes of a pilot study on trans-scleral iontophoresis delivery of lutein in patients with AMD. Topical delivery of lutein directly to the macula may provide a more efficient method for enriching the macular pigment and for achieving greater patient compliance to therapy than oral administration and thus enhancing prevention strategies. Modern diagnostic methodologies shall address the major problem of accurately detecting the risk of transition from intermediate AMD to advanced AMD stages. Adaptive optics retinal imaging and resonance Raman spectroscopy are two highly promising technologies for the objective assessment of patients with AMD. In this review, we present some of their clinical applications for collecting quantitative measurements of retinal cellular changes and macular content of xanthophyll pigments, respectively. In conclusion, there is great expectation that technological advancements in AMD management will deliver improved screening, therapeutic prevention, and diagnostic systems in the coming decade through a pro-active strategy of “treatment for prevention” that will aim to reduce the global burden of vision loss caused by AMD in the elderly.

## Introduction

An aging population is much more susceptible than younger people to many health problems, including eye diseases. The aging of the population in the world will experience a substantial increase in the size of the population aged 65 years or over by 2030, a population that accounts for the highest incidence and prevalence of eye diseases (1).

Age-related macular degeneration (AMD) is a chronic progressive disease and among the leading causes of low vision and legal blindness worldwide in people older than 60 years (2). Clinically, AMD is classified in early (Stages 1 and 2), intermediate (Stage 3), and late (Stage 4) stages according to the signs and symptoms complained by the subjects (3). Impairment of visual function starts in intermediate AMD and progresses to late AMD with vision-threatening complications like neovascularization (neovascular or “wet” AMD) or “geographic atrophy” (“dry” AMD) of the central retina (i.e., the macula). Late-stage AMD, the most severe form of the pathology, is present in about 5% of the over 65's and 12% of the over 80's (4, 5). According to the *Retinal Disease Panel of the National Plan for Eye and Vision Research* (6), AMD represents ~90% of cases of blindness in people aged 60 years or older. Vision loss caused by advanced stages of AMD has profound human and socioeconomic consequences in all societies. The costs of productivity loss and of rehabilitation for blindness constitute a significant economic burden for an individual, the family, and society globally (7–9). Of note, 30% of persons with advanced AMD also have clinical depression (10).

The focus of most research and development activities on AMD is aimed at evaluating the cost effectiveness of treatments of the neovascular forms. On the other hand, more effective strategies of secondary prevention for slowing down or halting AMD progression would be highly desirable. To be clinically efficient, such activities should require:

1) the identification of patients at higher risk of disease progression, and
2) the development of novel diagnostic technologies and treatment for preventing progression from intermediate to advanced AMD stages.

Addressing both issues can greatly benefit patients and society.

AMD is a multifactorial eye disease with several risk factors contributing to its onset and progression, such as genotype, aging, hypertension, hypercholesterolemia, smoking, overweight, and low dietary intake of vegetables, fish, and fruits. However, it has been shown that almost all patients with AMD could benefit from a healthy lifestyle, with those with a high genetic risk, showing the strongest risk reduction (10, 11). A high dietary intake of vegetables, fish, and fruits and no smoking have been shown to halve the risk of progression to late AMD in comparison with patients with an unfavorable lifestyle (11, 12). Current knowledge of the beneficial role of lifestyle on AMD progression shall drive clinicians and local authorities to more rigorous measures for prevention through behavioral change of populations. For example, high plasma concentration of xanthophyll pigments, such as lutein and zeaxanthin, has been associated with a 37% reduced risk to progress to late stage AMD in a prospective study (*Alienor study*) on 609 patients followed up to 7 years (13). Lutein and zeaxanthin accumulate physiologically in the macula and, by absorbing blue light, prevent the generation of reactive oxygen species (ROS) that can damage photoreceptor and retinal pigment epithelium (RPE) cells (14). Several clinical trials, including the *AREDS2, CAREDS*, and *Blue Mountain Eye Study*, have reported that regular and high dietary lutein and zeaxanthin intake reduces the risk of AMD progression (15–17). On the other hand, there are some barriers that halt clinicians to support prevention strategies that promote xanthophylls supplementation mainly caused by limits of current oral supplementation methodologies as well as of diagnostics technologies for measuring macular pigments. Efficacy of prevention strategies shall be assessed by sensitive and accurate diagnostic methodologies in order to measure objectively their beneficial action on disease progression along a clinically relevant time scale. For example, high-resolution ophthalmic devices for imaging the retina at the cellular level would be desirable for investigating pathologic tissue changes with micrometer accuracy and for tracking response to prevention strategies in advance with respect to current diagnostic tools. Adaptive optics retinal imaging has the potential to establish a novel methodology for screening patients at risk of AMD progression and to monitor the therapeutic effect of prevention strategies (18). In addition, diagnostic tools for measuring the xanthophyll pigments *in situ* would be desirable for elucidating and assessing the protective effect of prevention strategies based upon supplementation with lutein.

In this review, we provided the state-of-the-art knowledge about risk factors and prevention strategies for AMD and further presented former clinical outcomes on novel diagnostic and prevention methodologies for addressing one of the major needs in the management of AMD, which is to prevent vision loss in patients at higher risk of AMD progression.

## Global Impact of AMD

According to World Health Organization (WHO), more than 8 million people suffer from vision impairment caused by age-related macular degeneration (2); since AMD prevalence is directly related to age, and since the aging populations of the industrialized world are increasing rapidly, the impact of AMD on the socio-economical life in developed countries is expected to increase dramatically in the next years.

The prevalence and potential risk factors in late-stage AMD are similar across western and eastern countries, with higher prevalence in Chinese people than other ethnic groups (19). In a meta-analysis study (4), AMD has been estimated to be present in 0.2% of the population aged 55–64 years, rising to 13% of the population older than 85 years. Prevalence of neovascular AMD increases from 0.1% among subjects younger than 64 years to about 6% for those older than 85 years. Prevalence of pure geographic atrophy (GA) increases from 0.04 to 4.2% for these age groups. Based on the results of a systematic review (20), 30–50 million people suffer from any type of AMD globally. Conservatively, Europe accounts for 31% of global cases of AMD with a mean projected number of 17 million people that are living with AMD, of which 2.5 million cases have AMD Stages 3 and 4, and the number is expected to rise more than 30% by 2040 (21). A multinational study on AMD economic burden found that the average annual total cost for single patient with neovascular AMD varied from €5.300 in the United Kingdom to about €12.500 in Germany (9). Global cost of visual impairment due to AMD has been estimated to be €343 billion, excluding home health care costs and productivity losses (22, 23). A specific study estimating the productivity loss (i.e., loss of employment and loss of salary) caused by AMD has estimated that the total loss in gross domestic profit in the US due to dry AMD is, averagely, $24 billion (24). In the next decades, these values are expected to largely increase with projected demographic shifts (25). According to the United Nations predictions, the number of people aged over 60 will triple from 600 million worldwide in 2000 to 2 billion by 2050. The increase in the population aged over 80 is expected to be more than 5-fold, from 70 million in 2000 to 380 million by 2050 (26). In this context, the socioeconomic benefits of effective strategies for primary and secondary prevention of AMD could be enormous.

## Risk Factors

AMD is a multifactorial disease with numerous inherited and environmental risk factors contributing to its onset and progression. The non-modifiable risk factors include the inheritance of major genetic loci of AMD-associated genetic variants, local traits, such as darker iris pigmentation and hyperopic refraction, and aging (27–31). Overall, genes influence several pathological processes related to AMD, including the mechanisms involving collagen and glycosaminoglycans synthesis, angiogenesis, and the immune processes. All these factors have been associated with the onset and progression from early to intermediate, and advanced stages of AMD (27, 28). There are several known AMD-associated genetic variants (29), and some of them have been targeted by interventional clinical trials (30). The genetic contribution of the complement pathway (CFH, CFI, C9, C2, TMEM97/VTN, and C3 genes) and ARMS2 to AMD Stage 4 has been found to explain 90% of the overall genetic risk in a population of 17,000 patients (31). Gene therapy for AMD Stage 4 (either for treating “dry” or “wet” AMD) is, indeed, currently being explored. Several clinical trials are testing safety and efficacy of gene augmentation for endogenous production of soluble inhibitors of vascular endothelial growth factors (VEGFs), utilizing viral vectors delivered *via* an intravitreal injection. Genetic susceptibility is, however, influenced by the environmental factors; together, both factors can be highly predictive of the onset, progression, and response to treatments (32). In this view, strategies to minimize the influence and impact of environmental factors can greatly benefit to reduce the social burden of AMD. The modifiable risk factors include cardiovascular diseases, obesity, smoking, and sunlight exposure (33). Lack or poor physical activity rises the risk for several metabolic and vascular diseases and has been correlated with the progression of some cases of AMD (34). Several clinical trials have evidenced the beneficial role of nutrition (fish, fruits, and vegetables) and nutritional supplements (35). A healthy diet, avoiding food rich in sugar, fat, alcohol, and oils, was associated with reduced occurrence of early and/or advanced AMD (36). Absence of smoking and moderate physical activity (i.e., regular low-intensity exercise) have been also demonstrated to provide a protective role in AMD disease progression. In conclusion, the adoption of healthy lifestyles may benefit significantly populations, particularly those at genetic/family risk. Public health interventions promoting plant-rich diets, physical activity, and avoiding smoking and sedentary behavior would be highly recommendable strategies for AMD prevention. Current scientific and clinical evidence shows that supplementation with xanthophylls, lutein, and zeaxanthin can be of particular importance to prevent progression from early- to late-stage AMD (34). The protective effect of daily intake of several other supplements, which may have a role in slowing down disease progression, such as zinc (with xanthophylls), folate, curcumin, saffron, and goji berry, is under study (35). The protective role of beta-carotene, omega-3 fatty acids (DHA, EPA), vitamin A, vitamin C, and vitamin E against AMD progression has not been supported by epidemiological studies (14, 35).

## Prevention Strategies

### Antioxidant and Protective Effect of Lutein

Lutein is a dietary carotenoid from the xanthophyll family of carotenoids. Lutein and its isomer zeaxanthin are the main components of human retina's macular pigment. In the normal human retina, the concentration of these carotenoids is the highest across the foveal area, decreasing exponentially as distance increases from the fovea. The ratio of lutein to zeaxanthin is 1:2 in the macula and 2:1 in the peripheral retina; lutein is, therefore, 2.5–3 times more concentrated in the macula than the peripheral retinal region.

The main physiological functions ascribed to the macular carotenoids are:

1) a shielding effect protecting the retinal photoreceptor's membrane system against potentially harmful, short-wavelength radiation.
2) protection against photo-induced damage of the retinal photoreceptor's membrane system.

Nature has used xanthophyll pigments as an effective protector, capable of both absorbing damaging blue light and inhibiting the formation of ROS and neutralizing photosensitizers (37–39). The reason why xanthophylls accumulate selectively in these areas of the central retina is not yet fully elucidated. According to the most supported hypothesis, xanthophylls transversely incorporate in the lipid-bilayer portion of membranes of the human retina through xanthophyll-binding proteins (40, 41). These membrane-associated, xanthophyll-binding proteins bind lutein (and zeaxanthin) with high specificity and affinity.

The highest concentration of lutein and zeaxanthin is detected in the Henle fiber layer in the foveal region (2/3 of total) and in the photoreceptors' outer segment (1/3 of total) (42–44). The precise location of macular xanthophylls across the retina has been associated with specific functions aiming at protecting the retinal photoreceptors from oxidative damage (45, 46). The main physiological function ascribed to the inner retinal macular carotenoids is the protection of the retinal photoreceptors from photo-induced damage caused by harmful short-wavelength radiation by means of a shielding effect. The function of the outer retinal xanthophyll pigments as antioxidants and quenchers of ROS has been related to their physical interaction with the cell membrane lipid-bilayer and their membrane localization and accumulation in the bulk domain of the photoreceptor outer segment (POS) membrane.

Lutein absorbs blue light, with a maximum absorption peak at 460 nm (37, 47). This is the most phototoxic visible light to which the retina is routinely exposed, rendering lutein and zeaxanthin efficient physical quenchers of this harmful light, blocking the production of singlet oxygen and related ROS (38). Therefore, the oxygen deactivation property of lutein is a consequence of its ability to absorb blue light *via* the unconjugated double bonds present in the molecule. In addition, xanthophylls are selectively accumulated in the bulk domain of the POS membrane, which is rich in long-chain polyunsaturated fatty acids, including docosahexaenoic acid (DHA). Rhodopsin, which is the main protein of POS membranes (90% of all proteins in these membranes) and is responsible for the first stages of visual signal transduction, is also located in the POS membrane bulk domain. Rhodopsin requires the presence of polyunsaturated lipids for its activity; on the other hand, co-localization of rhodopsin with polyunsaturated phospholipids creates a dangerous situation for both, especially during illumination, when ROS are produced by photosensitizers (i.e., all-trans-retinal) (48). Such a selective accumulation of macular xanthophylls in domains rich in vulnerable unsaturated lipids is, therefore, ideal, given their photoprotective action. Reacting as antioxidant with free radicals and ROS, lutein protects the retinal photoreceptors against peroxidation and photo-damage.

Beyond acting as a blue-light filter and efficient quenchers of ROS, macular xanthophylls may also enhance vision contrast by reducing chromatic aberrations, glare disability, and light scattering (49–52).

According to current knowledge, the antioxidant and protective activities of lutein could be related to its effects on the physical properties of lipid bilayer membranes in the Henle fiber layer and the bulk domain of the POS in the foveal region (41, 45, 48, 50). Lutein is able to quench singlet oxygen by two different mechanisms. The first mechanism, which involves energy transfer, is called *physical quenching*. According to this mechanism, lutein deactivates singlet oxygen to the non-reactive triplet state. The second mechanism, which, however, contributes < 0.05% to the overall singlet oxygen quenching by carotenoids, is called *chemical quenching* and involves a chemical reaction between carotenoid and singlet oxygen, which results in pigment auto-oxidation.

### Oral Supplementation of Lutein

Lutein is not synthetized by the human body and can only be absorbed from a vegetable-rich diet. The daily dietary intake of lutein ranges from 0.5 to 4 mg in the western world (53, 54). Following normal dietary ingestion, the plasma lutein concentration ranges between 0.13 and 0.18 μM (i.e., between 0.07 and 0.10 μg/ml).

In several controlled epidemiological studies, dietary intake of lutein and its isomer zeaxanthin was associated with protection from risk of AMD progression. The *Eye Disease Case Control Study* has found the risk for advanced AMD was reduced by more than 40% in patients in the highest quintile of dietary carotenoid intake (> 6 mg/day) when compared to those in the lowest quintile (Odd Ratio, OR:0.57) (55, 56). The *Carotenoids in Age-Related Eye Disease Study* (*CAREDS*) concluded that lutein- and zeaxanthin-rich diets could protect against intermediate AMD in female participants <75 years of age (19). The *Blue Mountain Eye Study* reported that high dietary xanthophylls intake reduces the risk of AMD progression over 5–10 years (18, 57), patients in the top tertile of intake (≥1 mg/day) had a decreased risk of incident neovascular AMD, and those with above median intakes (743 μg/day) had a reduced risk of indistinct soft or reticular drusen when compared with the remaining population. In the *Age-Related Eye Disease Study* (*AREDS*), dietary xanthophylls intake (as determined by a food habit questionnaire at enrollment) was inversely associated with neovascular AMD (OR: 0.65), geographic atrophy (OR: 0.45), and large or extensive intermediate drusen (OR: 0.73) when the highest vs. lowest quintiles were compared (58).

An increase in dietary intake of lutein has been shown to raise its level in the plasma, which could provide a higher protection against photo-damage in human subjects at risk of AMD progression (16, 59). There is evidence that, after 1–2 months of daily supplementation, plasma levels of lutein stay at a higher level than the baseline while the supplementation continues; as supplementation is discontinued, plasma concentration of lutein decreases within 1–4 months to the pre-treatment level (60). Following 10-mg daily oral supplementation of lutein (the most common dose in commercial product), lutein plasma concentration has been shown to increase 3–5 times more than baseline values in healthy adults and subjects suffering from AMD (48, 60, 61). The increase of plasma lutein concentration has shown a significant correlation with the macular pigment optical density (MPOD), which has been estimated to increase to 5% (over a 1.5-degree area) in comparison with baseline measurements (Table 1). The MPOD is a measurement of the attenuation of blue light by macular pigments and is considered as an indirect measure of the amount of macular lutein and zeaxanthin in the macula.

**Table 1:** Clinical data on the effect of regular oral daily supplementation of lutein (10 mg).

**Baseline plasma concentration of lutein**	**Plasma concentration of lutein after 1–2 months of oral supplementation**	**Increase of Macular Pigment Optical Density (MPOD; 400 × 400 μm area) after oral supplementation**
0.13–0.18 μM (0.07–0.10 mg)	0.3–0.9 μM (0.17–0.5 mg)	5% greater than baseline

*AREDS2* study enrolled 4.203 participants, aged 50–85, with intermediate AMD in both eyes, or intermediate AMD in one eye and advanced AMD in the fellow eye. The main study outcome has shown that lutein and zeaxanthin intake resulted in a 10% reduction of progression to advanced AMD (Hazard Ratio; HR: 0.90; *p* = 0.04) (62). Further analysis has shown that the participants with low dietary intake of lutein and zeaxanthin at the start of the study, supplemented with an AREDS formulation, were 25% less likely to develop AMD Stage 4 than the patients with similar dietary intake who did not receive lutein and zeaxanthin (4). In a pre-specified comparison between patients with AMD receiving lutein/zeaxanthin vs. those who did not receive this supplementation, a 10% reduction in the risk for progression to late AMD has been recorded (4, 63).

Considering the overall supporting science on safety of dietary supplements of lutein (10–20 mg/day) for reducing the risk of progression from early to advanced AMD stages, supplementation may be a cost-effective approach for patients with AMD to reduce future impairment and disability. Nevertheless, compliance of patients to oral supplementation is still limited (64). This is mainly due to the type of administration and the requirement of daily intake of the therapy for prolonged time (theoretically, the supplementation should not be stopped). Another factor that significantly affects the efficacy of oral supplementation in increasing macular lutein content is the limited absorption of this carotenoid through the digestive route. Lutein is transported in the plasma *via* lipoproteins, primarily HDLs (52%) and, secondarily, by LDLs (22%) (64, 65). Lutein and zeaxanthin associate more closely with HDL, and it has been theorized that only a small proportion (2.5%) of HDL might be responsible for transporting lutein and zeaxanthin to the retina (66). The plasma concentration varies considerably among individuals and may be influenced by several factors involved in its absorption and plasma transport (type of lutein, duration of lutein intake, amount of fat in the diet, concomitant ingestion of fibers, genetic factors, age, etc.). In addition, it has been shown that substantial increase in macular pigments can be found only after at least 3 months of oral supplementation (38, 62, 67), so it would be important to implement new strategies to enrich the macular pigment faster than current mainstream method and to improve patients' compliance to therapy.

### Topical Delivery of Lutein

Iontophoresis is a non-invasive technique widely used in medical practice to deliver a charged molecule from a liquid formulation to a target tissue through the application of a low-intensity electric current (68). During iontophoresis, the current applied to an active electrode located in the ocular (either corneal or scleral) applicator flows to a passive electrode placed on the periorbital skin, thus promoting the movement of the charged liquid formulation and enabling the therapeutic molecule to penetrate in the ocular tissues (69).

Recent studies (70, 71) have provided preclinical and clinical data on a novel scleral iontophoresis device for delivering a lutein-enriched liquid formulation directly to the retina. The 0.1% lutein ophthalmic formulation was composed of FloraGLO^®^ crystalline lutein (Kemin Food L.C., Des Moines, IA, USA) encapsulated in positively charged liposomes using phospholipon 90H (Lipoid GmbH, Ludwigshafen, Germany), octadecylamine (Sigma-Aldrich, Saint Louis, MO, USA), and distilled water. The scleral iontophoresis device consisted of a generator, an applicator with the active electrode, which is filled with the lutein liquid formulation, and a return, passive, electrode. The generator's current was set at 2.5 mA and was delivered for a total 4 min.

In a first *ex vivo* study (70), two-photon microscopy has been used to quantify the amount of lutein, reaching the macular region in the human retina of eye bank donor eyes after scleral iontophoresis. Analysis of the two-photon emission fluorescence (TPEF) intensity signal collected in the fluorescence band spectrum of lutein was done in order to evaluate the increase of such a signal in samples that underwent iontophoresis in comparison with controls. Six eye globes, from different donors, were used for experiments, four of which underwent trans-scleral iontophoresis delivery of lutein and two eyes were used as controls. Details of study methodology can be found in Supplementary Material (72).

**Figure 1:**
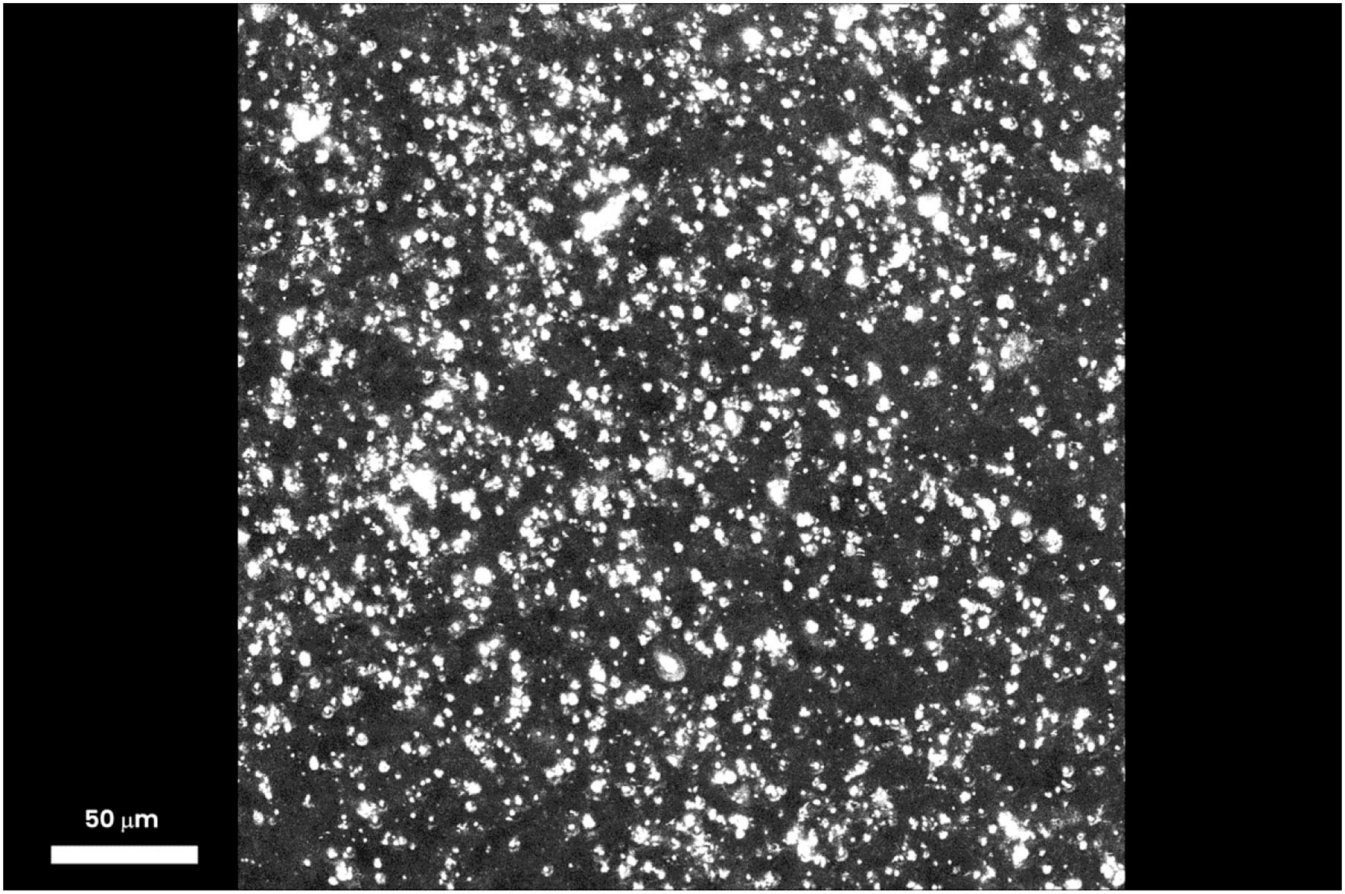
Three-dimensional projection stack of the macular area, showing several particles (≥5 μm), which emit a TPEF signal corresponding to lutein-enriched liposomes. One hour after trans-scleral iontophoresis, the lutein-enriched liposomes were spread throughout the outer and inner retinal depth; the major differences of the TPEF signal generated by exogenous lutein were found in the retinal layers between the photoreceptors and Henle's fibers. Scale bar: 50 μm.

**Figure 2:**
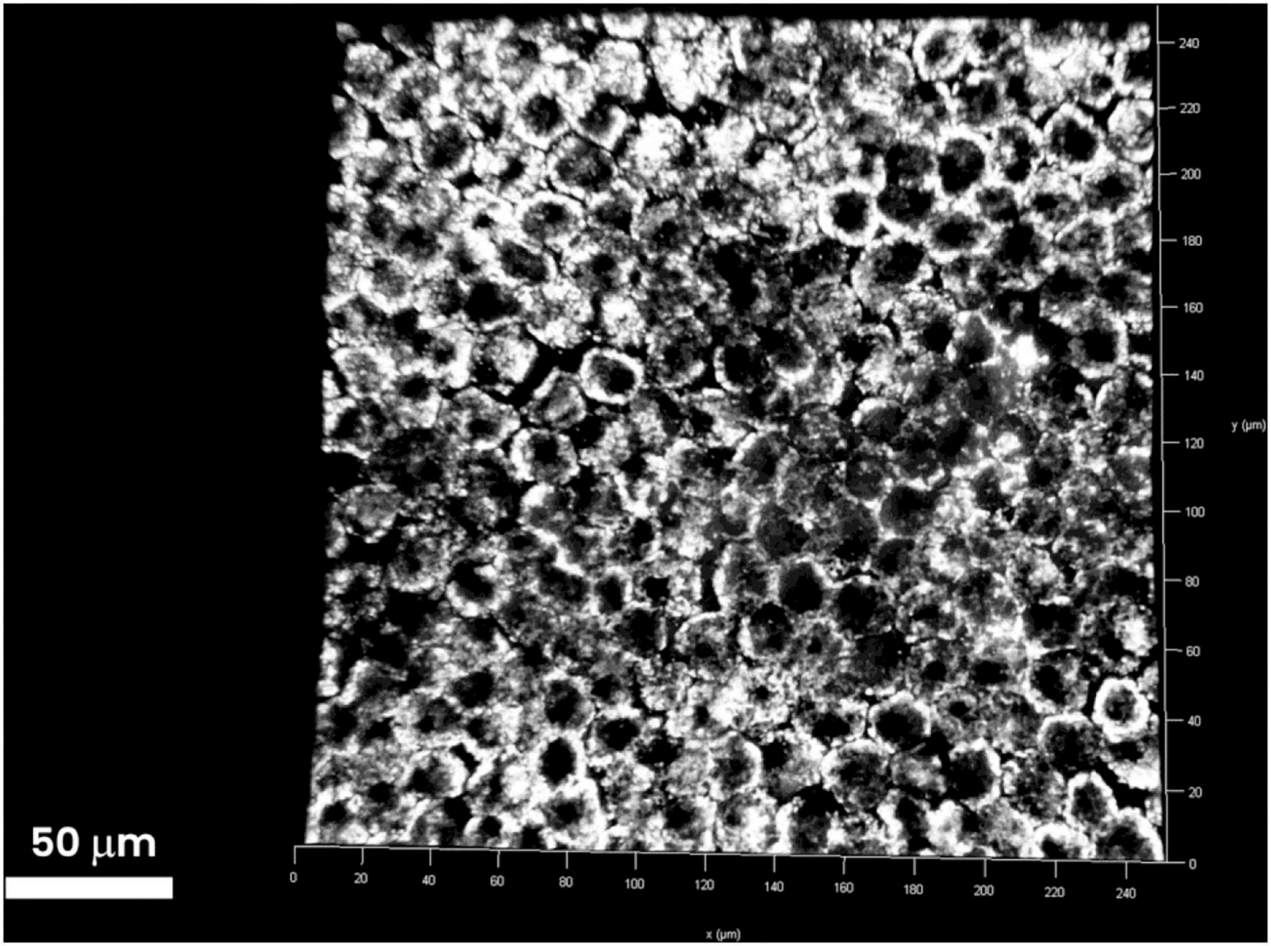
Three-dimensional stack of the RPE layer underlying the retinal periphery. RPE cells are tightly attached to one another and form a barrier between the choroid and retina. The RPE layer is increasingly impermeable to passive diffusion of molecules >400 Da. No extra-cellular particles emitting TPEF signals were found in the RPE layer. The TPEF coming from the intracellular content of RPE cells is generated by lipofuscin (1-μm particles). Scale bar: 50 μm.

**Figure 3:**
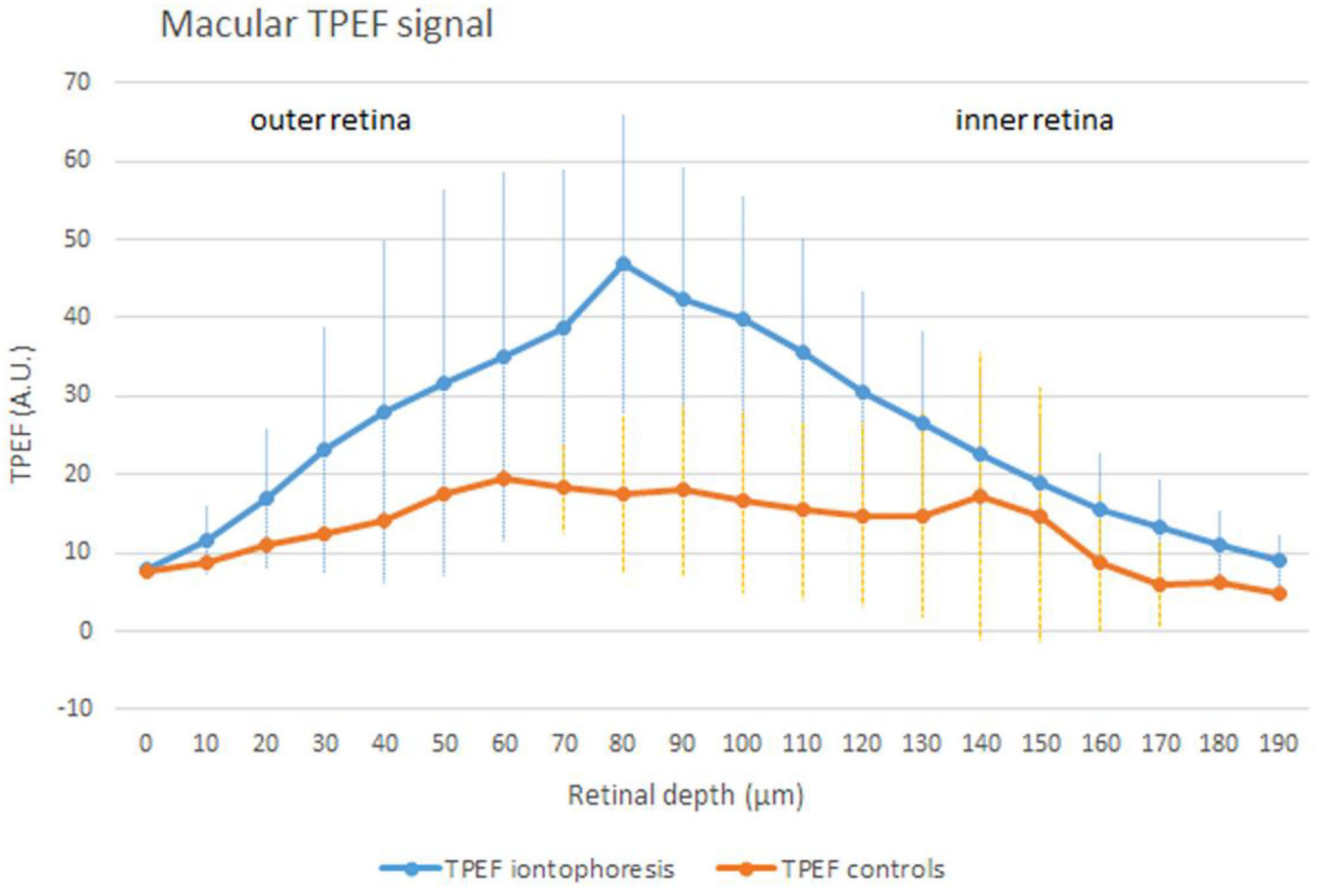
An average (± SD) TPEF intensity profile in samples treated by iontophoresis (the blue curve) and controls (the orange curve) in the macular area (500 × 500 μm). In eyes that underwent trans-scleral iontophoresis, the TPEF signal collected in the fluorescence band spectra of lutein was significantly higher than controls (*p* < 0.001). Across the inner retinal layers, the difference in the TPEF signal of treated and control eyes was still significant (*p* = 0.001); the inner layers are represented by the Henle's fibers at the fovea and the inner nuclear layer and the inner plexiform layer at the perifovea. In the normal macula, lutein is mostly concentrated in the photoreceptors' outer segments (<1/3 of total lutein; outer retina) and Henle's fibers (>2/3 of total lutein; inner retina). The excess of lutein in the macular region obtained after iontophoresis can represent the ideal local reservoir of the compound, which is selectively concentrated in the macula by retinal cells.

One hour after iontophoresis, features consistent with lutein-enriched liposomes were found both in the central and peripheral retina of treated eyes (Figure 1). Imaging of retinal pigment epithelial (RPE) cells and choroid did not show any lutein-enriched liposomes (Figure 2). A higher TPEF intensity level in the fluorescence band spectra of lutein was found in the macular region of treated eyes in comparison with controls (Figure 3); the greater differences between treated eyes and controls were found in the retinal layers between the photoreceptors and Henle's fibers. This was not surprising, because, in normal eyes, lutein is mainly concentrated in the photoreceptors' outer segments (both in the peripheral retina and macula) and the inner retina (only in the macula).

The concentration of lutein was estimated to be 21 μM in controls; this value was equated to the integral of the TPEF intensity signal of controls (i.e., the area under the orange curve in Figure 3). The increase of lutein after scleral iontophoresis was calculated as the ratio between the two areas, which were subtended by the TPEF signals of treated eyes (the area under the blue curve in Figure 3) and controls (the area under the orange curve in Figure 3). The concentration of macular lutein was 40 μM 1 h after iontophoresis, thus increasing the amount of lutein in the macula of 1.9 times in comparison with the baseline. The results are summarized in Table 2.

**Table 2:** Estimation of lutein concentration in the macular area of eyes after scleral iontophoresis of lutein and controls.

**Macular lutein in controls** **(*n* = 2 eyes)**	**Macular lutein in iontophoresis** **(*n* = 4 eyes)**	**Increase of lutein in macula (500 × 500 μm area) after iontophoresis**
21 μM (12 mg)	40 μM (23 mg)	90% greater than controls

In a second *ex vivo* experiment (71), resonant Raman spectroscopy (RRS) was used to confirm efficacy of scleral iontophoresis delivery of the positively charged lutein solution to the human retina in eye bank donor human eye globes. Resonance Raman spectroscopy is a vibrational spectroscopy technique that is commonly used to identify and quantify chemical compounds. Carotenoid molecules are especially suitable for Raman measurements since they can be excited with light overlapping their visible absorption bands (73, 74). When excited by blue light, these molecules exhibit a strong resonance Raman scattering response, enabling to detect their characteristic vibrational energy levels through their corresponding spectral fingerprint signature even in living human tissues.

In this study, a purpose-developed RRS was used for detecting lutein in human ocular tissues. Eight eye globes from different donors were used for experiments; six of which underwent trans-scleral iontophoresis delivery of lutein, and the remaining two eyes were used as controls. Details of study methodology can be found in Supplementary Material.

**Figure 4:**
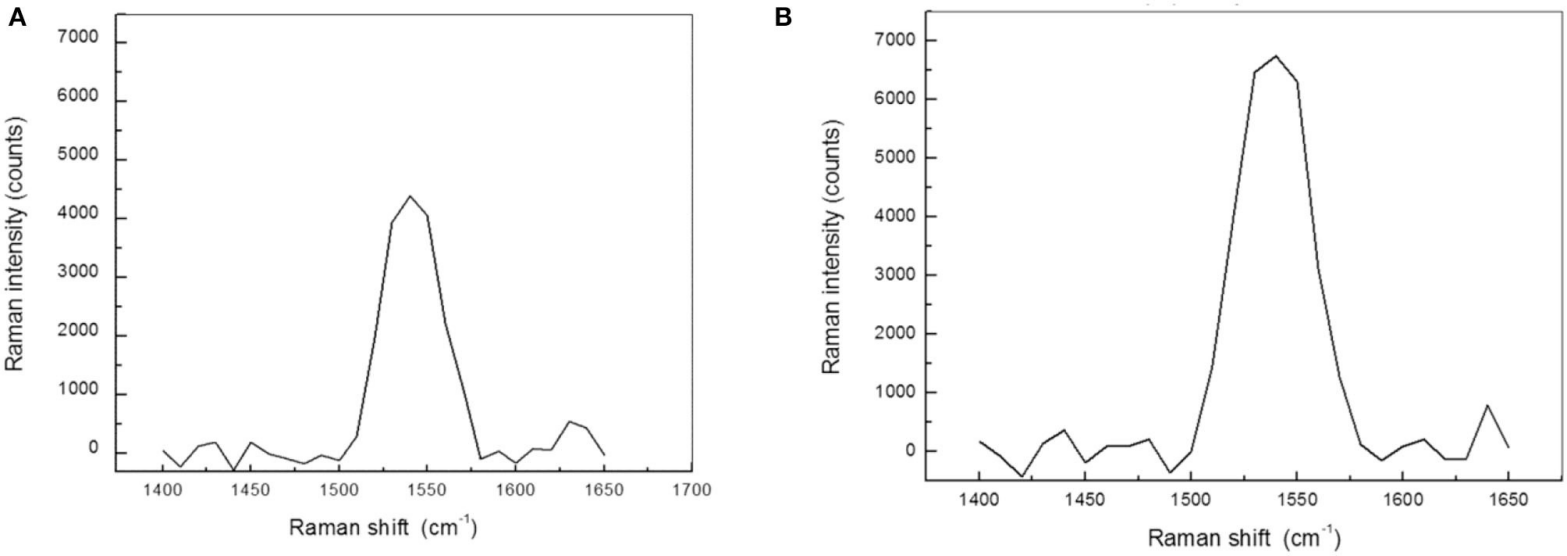
The macula shows the highest concentration of xanthophyll pigments in the retina of human eyes. **(A)** Resonance Raman spectra obtained from the macula of a control eye. The Raman peak is specifically related to xanthophyll pigments. **(B)** Resonance Raman spectra of the macula 1 h after transcleral iontophoresis with 0.1% lutein ophthalmic solution. The concentration of macular pigments in treated eyes was, on average, 25% higher than control eyes (*p* < 0.05).

One hour after iontophoresis, the inner sclera, choroid, and retinal periphery were greatly enriched with lutein in treated eyes (*p* < 0.05); no lutein was found in the same ocular regions of non-treated samples. In the same period, the average concentration of lutein in the macula of treated samples was 1.3 times greater than controls (Figure 4). The results are summarized in Table 3.

**Table 3:** Concentration of lutein in ocular tissues (ng/mm^2^, M ± SD).

	**Inner sclera**	**Choroid**	**Peripheral retina**	**Macula**
Controls (*n* = 2)	0	0	0	3.7 ± 1.0
Iontophoresis (*n* = 6)	16 ± 9.0*	1.2 ± 0.3*	2.5 ± 1.3*	4.8 ± 1.7

* *P < 0.05 between study and control groups*.

In conclusion, both *ex vivo* studies on eye bank human donor eyes have provided clear indication that scleral iontophoresis of a positively charged lutein liquid formulation could be effective in enriching the macular pigment of the human eye in few minutes.

### Pilot Clinical Study on Scleral Iontophoresis Delivery of Lutein

**Figure 5:**
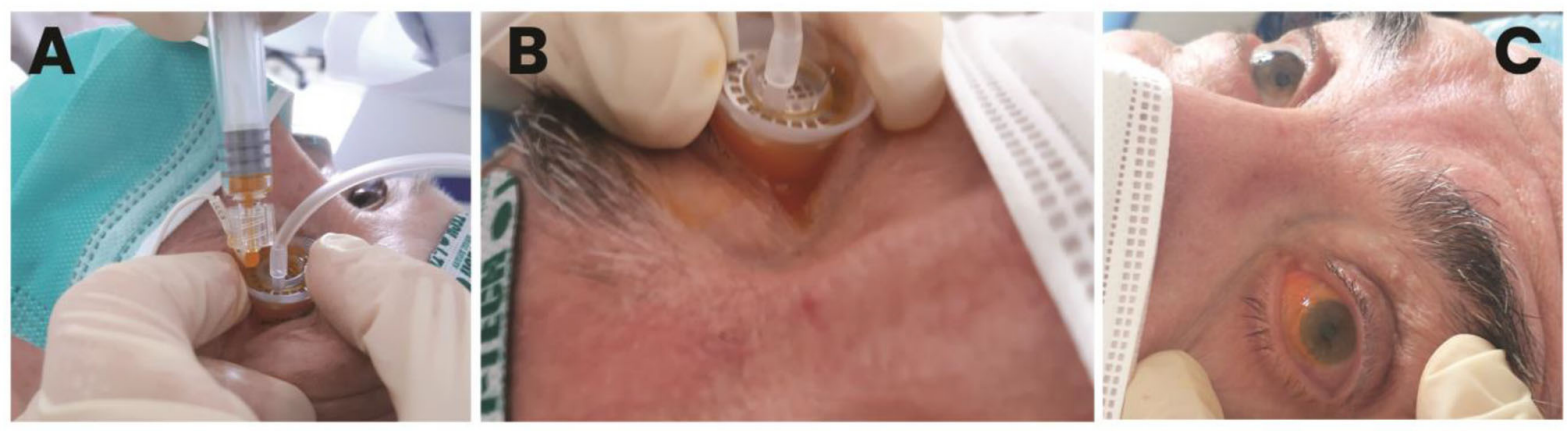
Main treatment steps of trans-scleral iontophoresis delivery of 0.1% lutein ophthalmic solution. After placing the passive electrode onto the patient's forehead and connecting the electrodes to the generator, the active electrode is filled with the lutein formulation **(A)**. Electric current is set at 2.5 mA for 4 min **(B)**. At the end of treatment, the ocular surface is gently washed with balanced salt solution to remove the excess of lutein; the patient is asked to rest onto the operating bed for 5 min **(C)**.

A pilot study was performed by the present authors to confirm safety and to assess tolerability in patients with AMD. The study followed the tenets of the declaration of Helsinki, and all the patients signed written informed consent after full explanation of the procedure. The study was granted exemption because no investigational method or device was used on patients (i.e., the scleral iontophoresis and the lutein liquid formulation were already CE certified before commencing the study). The inclusion criteria were the patients older than 40 years old, both genders, with a diagnosis of AMD with drusen (Stages 2 and 3) or neovascular AMD (Stage 4) in either eye and a corrected distance visual acuity (CDVA) ≤ 0.1 LogMAR. AMD severity was classified using *Age-Related Eye Disease Study* (*AREDS*) criteria (75). The exclusion criteria included heavy smokers (more than 20 cigarettes per day); pregnancy; presence of corneal scars or cataract; glaucoma; dry eye syndrome, Stage 4; and any additional eye disease other than AMD. Assessment of safety of scleral iontophoresis of lutein was determined by assessing CDVA using the EDTRS chart and central retinal thickness (a 1-mm ETDRS sector) using Optical Coherence Tomography (Canon HST-100, Japan) at the baseline and 1 week, 1 month, and 3 months after treatment. In addition, the patients were asked to fill a patient outcome report for self-assessment of ocular itching, lacrimation, photophobia, and ocular discomfort at 1 week postoperatively. At the baseline, each patient received one application of scleral iontophoresis, which consisted of (1) applying a drop of anesthetics (0.4% oxybuprocaine, Novesina, Novartis, US) onto the eye to treat and the return electrode onto the forehead after cleaning the area with 70% alcohol; (2) connecting the power supply battery (K-IONO, Offhealth SpA, Italy) to the ocular applicator *via* a cable; (3) applying the ocular application to the eye to treat and fill it with the lutein liquid formulation (Lipo+, Offhealth SpA, Italy); (4) setting the current intensity at 2.5 mA for 4 min. At the end of the procedure, after removing the ocular application and rinsing the eye with balanced salt solution, the patient was invited to rest lying down on the operating bed for 5 min. The main treatment steps of scleral iontophoresis of lutein are summarized in Figure 5.

Clinical data were summarized as mean and standard deviation; the Wilcoxon test was used to compare baseline and postoperative values at each examination interval. Statistical significance will be set at 0.05. All the analysis will be performed using the statistical software SPSS.

Nine (*n* = 9) patients, with mean age, 69 ± 9 years (range; 57–73 years; six females and three males), were enrolled in this pilot study. Three patients had diagnosis of AMD Stage 2, three patients had diagnosis of AMD Stage 3, and three patients had diagnosis of AMD Stage 4. The clinical outcomes of the pilot study are summarized in Table 4. The CDVA improved significantly from 0.20 ± 0.19 LogMAR to 0.11 ± 0.18 LogMAR (*p* = 0.01) at 1 week postoperatively, and then returning toward preoperatively value during follow-up. The 1-mm EDTRS sector retinal thickness did not change during follow-up (from 263 μm at the baseline to 262 μm at 3 months; *p* = 0.52).

**Table 4:** Clinical data outcome of the pilot study on scleral iontophoresis delivery of lutein.

	**CDVA (LogMAR)**	**ETDRS letters (n.)**	**1-mm ETDRS sector (μm)**
Baseline	0.20 ± 0.19	45 ± 10	263 ± 32
1-week	0.11 ± 0.18*	50 ± 10*	263 ± 32
1-month	0.13 ± 0.22	50 ± 11*	263 ± 32
3-months	0.16 ± 0.19	48 ± 10*	262 ± 32

* *p < 0.05 between post-operative and baseline values*.

The patients did not complain of pain or discomfort during the treatment. At 1 week, the patient outcome report score for tolerability was low for all symptoms, as summarized in Table 5. Overall, the clinical data from the pilot study demonstrated that scleral iontophoresis for topical delivery of lutein is safe and well-tolerated; randomized controlled clinical studies will be helpful to support evidence on efficacy of this novel secondary prevention strategy in patients with higher risk of AMD progression from early to advanced stages.

**Table 5:** Patient outcome report on tolerability.

	**Ocular itching**	**Lacrimation**	**Photophobia**	**Ocular discomfort**
Patient 01	1	0	0	1
Patient 02	0	0	0	1
Patient 03	1	1	0	0
Patient 04	0	1	0	0
Patient 05	1	0	0	1
Patient 06	0	0	0	0
Patient 07	1	0	0	1
Patient 08	0	1	0	1
Patient 09	1	0	0	1

*For each symptom, score ranges from 0 (any symptom) to 4 (highest intensity for the symptom)*.

## Novel Diagnostics

In the last decade, ophthalmic imaging has advanced from simple photography of the eye disease to an advanced investigation tool, enabling clinicians to better understand disease phenotype and to make quantitative assessments of the eye.

AMD is usually investigated by assessment of visual acuity, optical coherence tomography (OCT), and retinal/choroidal angiography using fluorescein/indocyanine green dyes. Currently, clinical diagnosis of established cases of AMD is evident on clinical examination when gross macroscopic alterations occur at the retina, such as drusen larger than 65 μm, hemorrhages, serous or hemorrhagic retinal or RPE detachment, RPE atrophy etc. With the advent of the anti-VEGF therapy, OCT retinal imaging has been increasingly used for the early diagnosis of choroidal neovascularization (CNV) and for the treatment and re-treatment management. OCT imaging is currently the paradigm of the diagnostic procedure to diagnose retinal diseases; in particular, analysis of SD-OCT and OCT-angiography retinal images has shown to provide clinically significant information on both disease progression and functional outcomes after anti-VEGF treatment (76, 77). Nevertheless, OCT is not sensitive enough to identify early signs or those patients with higher risk of disease progression from intermediate to late AMD stages because of relatively poor spatial resolution and lack of intrinsic functional information from retinal and RPE cells and retinal/choroidal microvasculature. There is, therefore, need to develop reliable methods for detecting specific signs of disease progression in the retinal tissue in a much shorter time frame than current state-of-the-art technologies.

The prerequisite for successful patients care in this age-related disease area is the development of diagnostic devices to facilitate definition of new and appropriate label-free clinical endpoints with high sensitivity and specificity. Such endpoints need to be meaningful to clinicians and patients and not limited to visual acuity, which is, currently, the only generally accepted functional clinical endpoint in retinal diseases, although it does not have enough sensitivity to reliably and consistently monitor disease progression. There is need for objective and sensitive clinical endpoints, assessing both the structural (e.g., drusen volume and characteristics) and functional (e.g., photoreceptors reflectance, choriocapillary blood flow) macular abnormalities. In addition, the ideal clinical outcome metrics should be able to establish sub-phenotypes of intermediate AMD with high risk to develop late-stage AMD and to early detect the response to therapy. Such assessments could greatly contribute to reduce the incidence of AMD progression from intermediate to late AMD stages. For example, progression from early AMD to the advanced stage of AMD has been related to a set of signs, such as the number and type of (soft) drusen in the macula, drusen size, the presence of pigment irregularities in the macular, age > 65 years, previous cataract surgery, cigarette smoking, and family history of AMD (mostly due to genetic variants of CHF and ARMS2 genes). More knowledge of additional and, possibly, more specific risk factors of AMD progression, such as those related to the macular xanthophyll pigments, would also contribute to improve more appropriate patients' management and care.

Among the novel diagnostic tools, adaptive optics ophthalmic imaging can resolve the retinal microscopy *in vivo* with unprecedented spatial resolution for detection of details smaller than 3 μm, and resonance Raman spectroscopy is a promising technology for the measurement of macular carotenoid levels in the living human retina.

### Adaptive Optics Retinal Imaging

It has been clear for long time that, by the time pathology is visible with current imaging tools, significant neuro-retinal cellular damage has already occurred. The resolution at which retinal images could be recovered *in vivo* by current retinal cameras is limited to the macroscopic scale (lateral resolution is ≥ 12 μm). In order to bring the lateral resolution of ophthalmoscopes to the microscopic scale (i.e., lateral resolution ≤ 4 μm), it is necessary to compensate both for low- and high-order optical aberrations of the eye.

Adaptive optics (AO) is a technology used to improve the performance of optical systems by reducing the effects of optical distortions. It provides considerable improvements in the contrast and sharpness of retinal images that are normally degraded by ocular aberrations when combined with any one of the known imaging modalities (e.g., fundus camera, SLO, OCT) (18). The benefit of AO for high-resolution retinal imaging has been clearly shown in numerous reports with the discovery of differences in the pattern of the cone/rod mosaic in various diseases, including AMD, diabetic retinopathy, inherited retinal dystrophies, and glaucoma (78).

Adaptive optics retinal imaging systems have demonstrated the capability to resolve numerous microstructural aspects of the living human retina. They make possible to resolve photoreceptor cells, including cones and rods, retinal nerve fibers and microvasculature; the improved resolution provides a more sensitive tool with which to study, detect, and track retinal diseases (78). Given the prevalence of AMD, it is likely that this will be one of the more active growth areas in clinical AO imaging. The availability to combine complementary AO imaging, such as *en face* and axial scanning of the retina, and detection, such as bright- and dark-field modality, options at the same time can provide a holistic approach to in-depth investigation of the retinal photoreceptors, capillaries, and nerve fiber bundles.

In early stages of AMD (i.e., stages from 1 to 3), the ability to predict the rate of progression is currently limited. By monitoring drusen over time, *en face* and axial AO imaging could be used to monitor drusen progression in terms of number and size, and assess their direct effect on the overlying photoreceptor mosaic (79). Preservation of cones over the drusen (either large colloid drusen or basal laminar drusen) could be clearly observed in patients (79). In a study of early AMD, the authors identified several additional small drusen deposits that were not observed with wide field fundus imaging or SD-OCT in early AMD stages (80). AO-SLO imaging also revealed a decrease in photoreceptor density and increased cone spacing in patients with AMD Stages 1 to 3, as well as a spectrum of photoreceptor changes, ranging from variability in cell reflectivity to decreased cell density (81). The increased reflectivity of photoreceptors associated with the drusen could be attributed to increased scatter from the RPE (due to decreased melanin or accumulation of some waste material) and to loss of outer segment pigments or loss of the photoreceptor outer segment. Significant decrease in visible photoreceptor density was also found in large coalescent drusen and areas of GA in advanced stages of dry AMD. A 30% decrease in cone counts was found (at 5–7 degrees eccentricity) in eyes with later stages in comparison with eyes with earlier stages of AMD progression. In addition, AO imaging has been shown to visualize reliably disruptions to the photoreceptor mosaic even outside the clinically visible GA lesions and to track the progression of the GA lesions over time (82). As such, a sensitive, non-invasive, high-resolution imaging tool could help to better recognize the earliest retinal changes and to identify patients who could progress rapidly and may benefit from a more intensive observation and management (83, 84). Furthermore, a better diagnostic approach of the macular disease could have an important role in the evaluation of the effectiveness of new prevention strategies at the cellular level.

**Figure 6:**
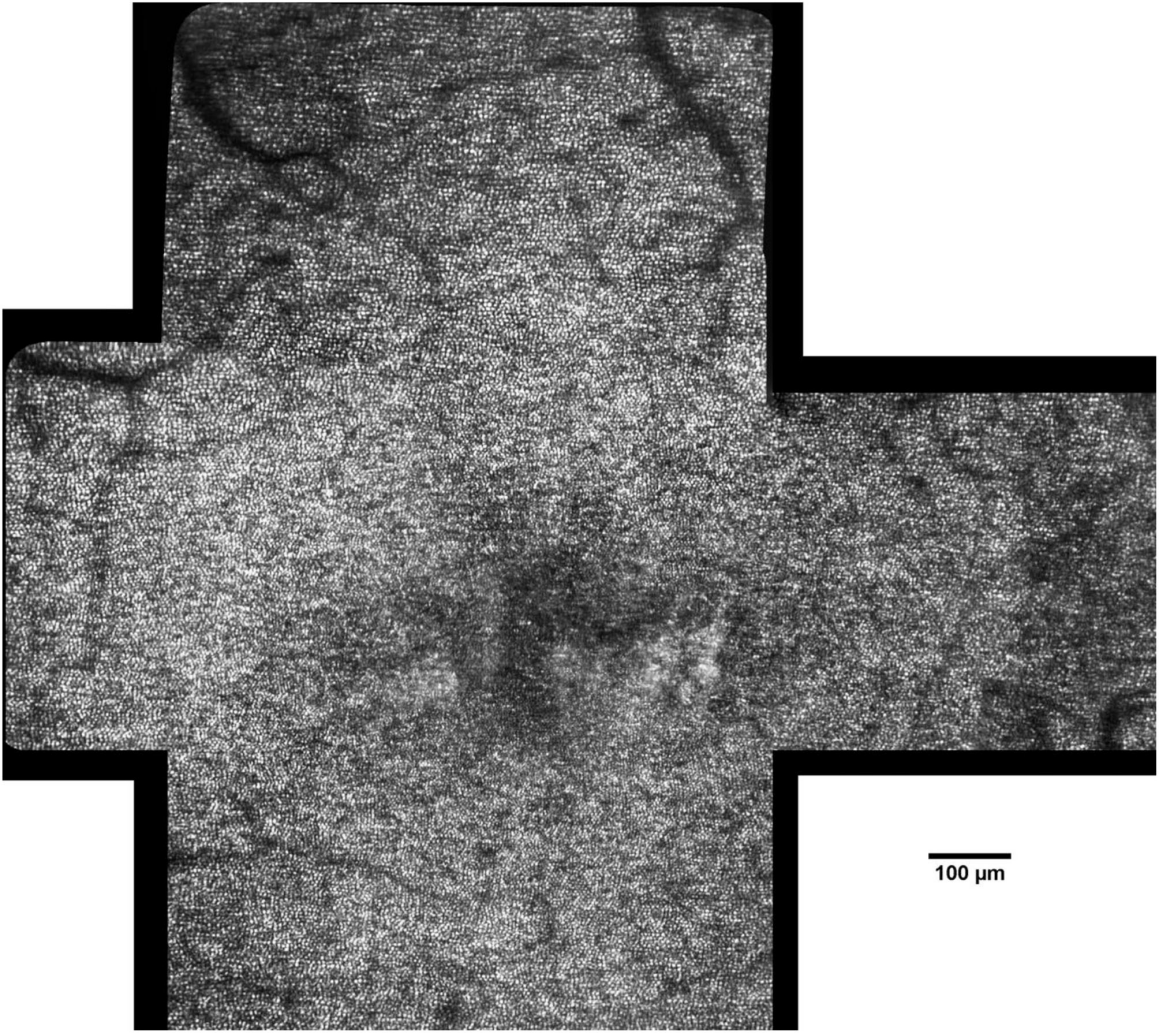
Montage of AO images in a 45-year-old female patient (scale bar: 100 μm). The photoreceptor mosaic can be clearly resolved across the central retina. Image processing and analysis of AO retinal images allow the clinicians to detect and track pathologic changes of the cone mosaic with cellular resolution.

**Figure 7:**
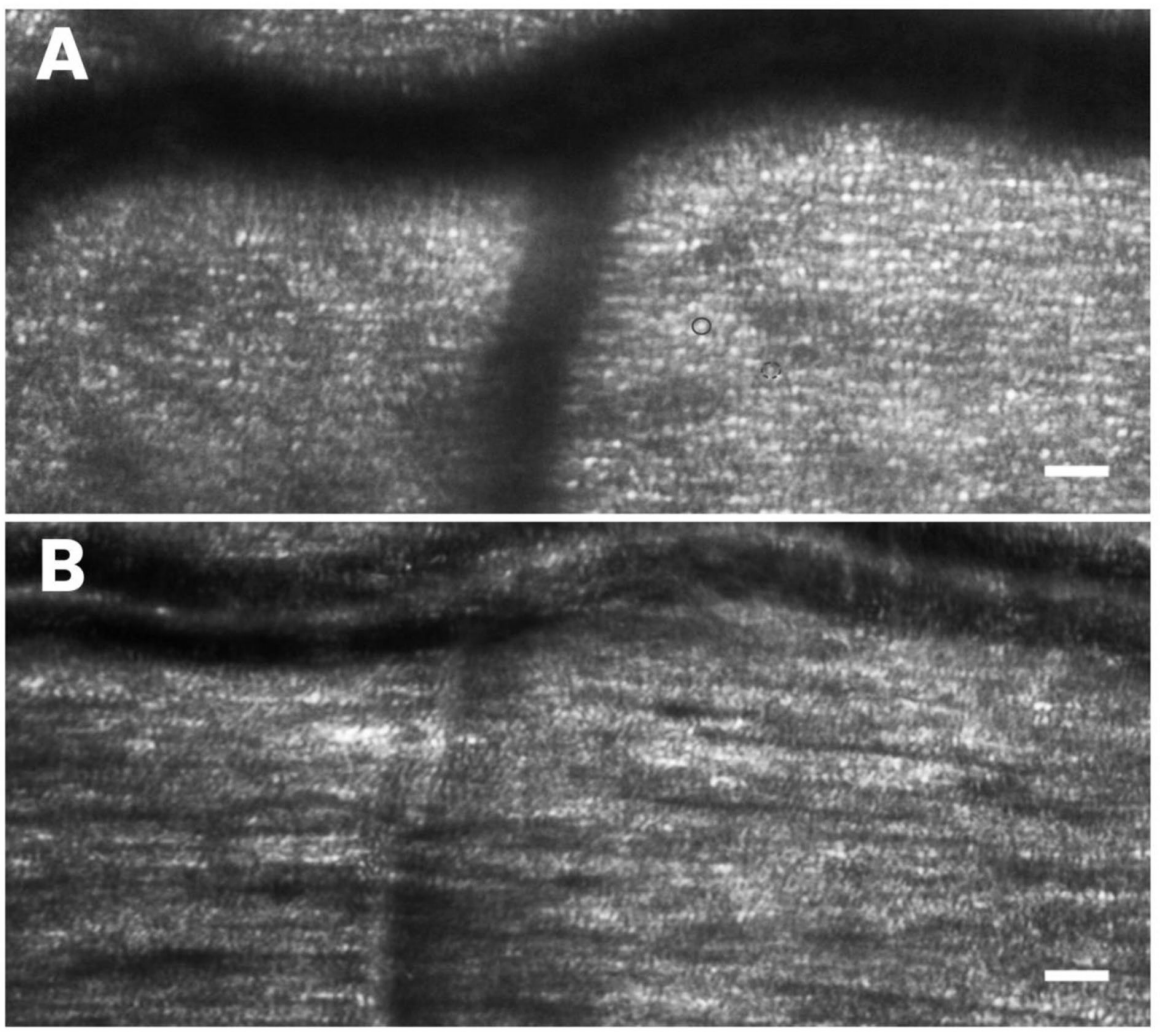
High-resolution AO imaging allows the clinicians to scan the retinal microstructures easily for rapid screening of health and integrity of the outer and inner retinal layers. In **(A)** the photoreceptor mosaic, including cones (brighter and bigger dots; the full circle) and rods (dimmer dots surrounding the cones; the dotted circle), is shown. In **(B)** the nerve fiber bundles, overlapping the cell mosaic is well-resolved. Scale bars: 50 μm.

**Figure 8:**
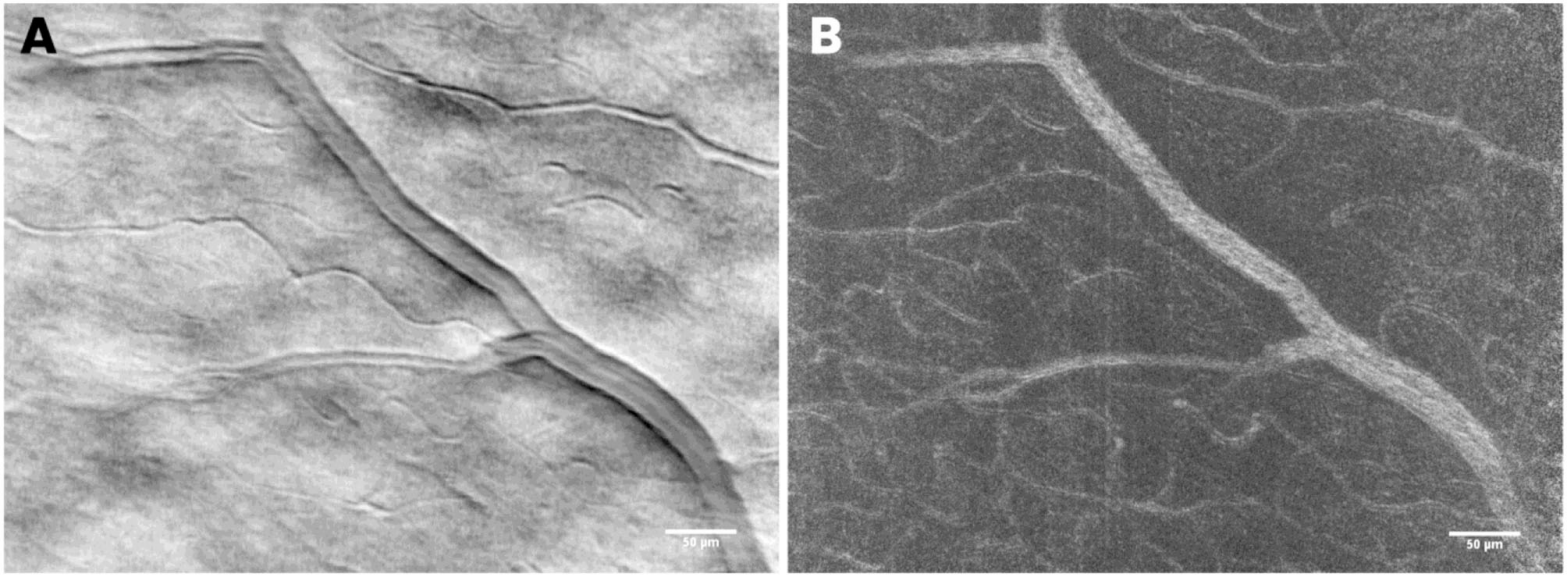
In the dark-field detection mode, retinal vascular structures can be resolved with high resolution. The use of a time-domain integration (TDI) line camera allows the user to exploit the split field imaging modality, which consists of collecting two shifted images on the camera, subtracted and divided by their sum. In **(A)** the mean TDI image and in **(B)** the standard variation, TDI image showing details of the retinal vessel walls and beds, respectively. Scale bars: 50 μm.

**Figure 9:**
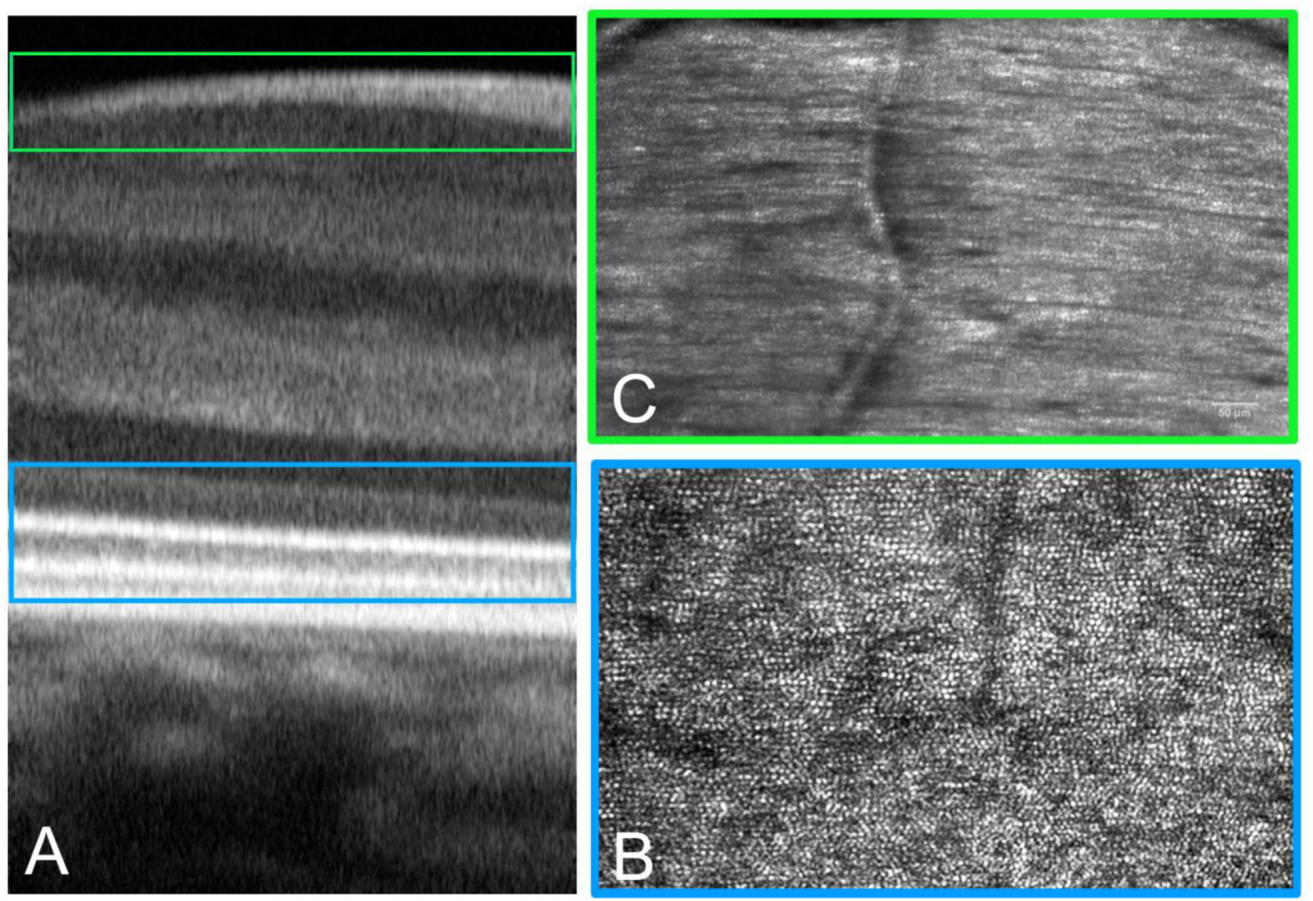
Adaptive optics technology can be implemented in a multimodal imaging system to acquire images of the retina, showing details of the tissue with histologic resolution *in vivo*. Multimodal imaging provides spatial co-localization of complementary information on structure (and function). Cross-sectional **(A)** and *enface* **(B,C)** AO images of the retina. **(A)** The AO-OCT scan provides detailed information on tissue layers from the choriocapillaris to the inner limiting membrane. **(B)** The same retinal area scanned at the IS-OS layer showing the photoreceptor mosaic. **(C)** High-resolution images of the retinal nerve fiber bundles are acquired with the polarizer oriented for maximum through output (paralleled to the major axis of bundles).

These authors tested different AO imaging modalities *in vivo*, providing former clinical data on patients using either flood illumination or line scanning laser ophthalmoscopy (LSO) imaging modalities (18). The advantage of the line scanning approach is that it multiplexes the illumination and detection in one dimension, while rejecting scattered light outside of the confocal range gate defined by the pixel size in a similar fashion as a flying-spot SLO; nevertheless, the line scanning approach reduces the hardware complexity by elimination of a high-speed scanner and associated mirror or lens relays. A multimodal AO-LSO-OCT system (PSI Corp., MA, USA) has been operated in both bright-field and dark-field detection modalities for imaging the retina. In the bright-field mode, quasi-confocal imaging was obtained by illuminating the retina with a scanning light source, which is collected by a line-array CMOS sensor for high contrast imaging of the retinal photoreceptors. Using this mode, images of the photoreceptor mosaic could be collected across the central and peripheral retina at high resolution; even the foveal cones within 1 degree from the foveal center could be clearly visualized (Figure 6). Polarized detection could be achieved within the bright-field modality by illuminating the eye with linearly polarized light and by filtering the light exiting the eye with a linear polarizer (or analyzer). Images of the retinal nerve fiber layer (RNFL) bundles could be acquired with the polarizer oriented for maximum through output (i.e., parallel to the major axis of bundles), and then at 45° and 90° relative to that orientation (Figure 7). In the dark-field detection mode, the light back scattered from the retina is collected by a time-domain integration (TDI) line camera in order to improve resolution of acquired images of the retinal vascular structures (Figure 8). High-resolution OCT could be performed simultaneously during LSO imaging of the retina (synchronous image acquisition). The complementary AO-OCT imaging modality does offer additional technical advantages compared to AO-LSO, providing a high-resolution cross-sectional view through the living retina, comparable to a histological section (Figure 9) (85).

The possibility to analyze retinal photoreceptors in AO images allows for assessing their density and arrangement as well as their waveguide and reflectance properties. Clinical studies have demonstrated how AO technology is sensitive enough to detect retinal tissue abnormalities with unprecedented spatial resolution, providing clinical information that is invisible to a current diagnostics tool (86, 87).

There are currently few barriers to a wide adoption of AO technology in clinical ophthalmology: the main obstacles are the high cost of sensing and correcting elements and the system complexity. A user-friendly AO instrument that can be used by ophthalmologists will facilitate the introduction of this technology into clinical practice.

### Resonance Raman Spectroscopy

Although the protective effect of dietary lutein/zeaxanthin on slowing down progression and severity of AMD is under intensive investigation, there is no commercially available diagnostic tool able to detect the amount of xanthophyll pigments in the macula and to monitor for their effect on preventing AMD progression from intermediate to advanced stages in response to dietary intake or emerging topical administration.

Resonance Raman spectroscopy (RRS) is one of the most promising technologies for the measurement of macular carotenoid levels from the human retina (71). It is precise, sensitive, specific, reproducible, and objective. It has also the potential to be translated to clinical applications in order to collect quantitative, direct, measurement of macular content of xanthophyll pigments.

To date, the macular carotenoid levels could be measured only indirectly using the macular pigment optical density (MPOD) measurement (88). There is a variety of methods currently in use that claim to measure the MPOD *in vivo*. Technically, the MPOD is a measurement of the attenuation of blue light by macular pigments and could be somewhat indirectly related to the amount of lutein and zeaxanthin in the macula; however, uncertainties exist on whether the MPOD may be reliably correlated with the amount of xanthophyll pigments in the retina. The methods to measure MPOD are divided into psychophysical (sometimes referred to as “subjective”) and physical (sometimes referred to as “objective”). The psychophysical techniques available include heterochromatic flicker photometry and color matching; the physical techniques include Fundus AutoFluorescence (FAF) and fundus reflectance. Validation of macular pigment subjective measurement techniques is still the subject of lively debate, and, due to their intrinsic technical limits, these methodologies are not gaining popularity among ophthalmologists and retinal specialists. Current physical methods are based on a variety of assumptions and are vulnerable to several technical limits. These methods shall (1) take into account single- and double-path light traversal through deeper retinal layers, (2) eliminate image contrast, diminishing fluorescence and scattering from the optical media (*via* confocal detection techniques, filtering, etc.), (3) bleach the photoreceptors, and (4) use a location in the peripheral retina as a reference point. For these reasons, auto-fluorescence and reflectance patterns hold poor specificity and are not sensitivity enough to follow AMD disease progression.

Contrary to all existing methods, RRS has no assumptions other than approximating the spectrally broad background fluorescence with the fluorescence response at a wavelength that is slightly offset from the macular xanthophylls Raman response (71). It directly measures absolute amounts of carotenoids in the illuminated region; in addition, it provides a measure of the absolute xanthophylls concentration distribution in the macular region since it does not use a reference point in the peripheral retina.

Resonance Raman detection of xanthophyll molecules is highly specific and can be established as a label-free tool for reliably evaluating the amount of macular pigments in patients. Xanthophyll molecules have been reliably detected in the living human retina (89–91), and the Raman signature of the xanthophyll molecules has been shown to be identical to the isolated carotenoid molecule. The authors have demonstrated feasibility and safety of RSS of macular xanthophylls on excised retina and human subjects, showing different patterns of macular pigment distribution (peaked, plateaued, ring shaped) (71, 89, 92, 93). It remains to be understood how the technique could be translated in clinical practice for monitoring and tracking the concentration of macular xanthophyll pigments in patients at risk of AMD progression. Overall, the advantages of RSS can be summarized in the following features:

- *objective measurement*: resonance Raman spectroscopy is a non-invasive technology able to directly measure and monitor the concentration of macular xanthophyll pigments in the living human retina;
- *molecular contrast*: excitation of the retina with a blue laser source enables the generation of a characteristics resonance Raman scattering response from xanthophyll molecules and allows for their accurate measurement from their corresponding spectral fingerprint signature in the living human retina;
- *spatial mapping*: scanning the blue light over the central retina allows for depicting and tracking over the same area a bi-dimensional map of the macular xanthophyll pigments content in the living human retina.

The limit consists in the implementation of a quantitative, reliable, method for data analysis and for eliminating the influence of the crystalline lens absorbance, which increases with aging and varies among populations.

## Conclusion

Age-related macular degeneration is the major eye disease in the aging populations worldwide. Knowing that the total cost savings derived from avoided AMD events for the same population is, on average, €4 billion per year, both novel diagnostic approaches aiming at identifying patients at risk of AMD progression and effective secondary prevention strategies to halt disease progression would greatly benefit society worldwide. Currently, the most indorsed pathophysiologic pathway of AMD includes a relationship with genotype, age, and lesions to the photoreceptor/RPE/Bruch's membrane/choriocapillaris complex, which have been associated with light-induced oxidative damage (*blue light hazard*). Systemic risk factors, such as smoking, hypercholesterolemia, overweight, and cardiovascular diseases, likely contribute to increased oxidative stress in the choriocapillaris complex. A healthy lifestyle, including a type of diet rich in vegetables, fruits and fish, and regular exercise are recommended to counteract the effect of aging and oxidative stress. In this context, there is increasing evidence that xanthophyll pigments may play an important role in maintaining the health and function of the macula. Their levels are known to decrease with aging and are significantly lower in patients with AMD, making the retina more vulnerable to light-induced damage with advancing age. Several clinical trials, including the *CAREDS*, the *Blue Mountain Eye Study*, and the *AREDS2*, have reported that higher dietary lutein and zeaxanthin intake reduces the risk of AMD progression. On the other hand, there are some barriers that halt clinicians to support prevention strategies that promote xanthophylls supplementation, mainly due to the low patient adherence to therapy and the limits of current diagnostics technologies for measuring macular pigments. New prevention strategies may involve topical delivery of lutein directly into the eye in order to allow clinicians to test directly the effect of xanthophylls on slowing down or halting disease progression in patients at higher risk. On the other hand, because the rate of disease progression is typically slow, it is estimated that patients have a clinical loss of visual function years after the onset of early signs of AMD. For this reason, both the early diagnosis and the monitoring of treatment efficacy at a cellular level represent two of the greatest needs in the management of AMD. If AO imaging succeeds in entering the clinical practice to provide high-resolution, objective, measures of the cone photoreceptor structure, RPE, and micro-vasculature in patients, it will open a new frontier for establishing effective patient care with novel prevention and treatment strategies.

Efforts will be also needed for improving management of patients suffering from AMD Stage 4. Although gene therapies have potential and are promising, there are significant obstacles, such as safety, long-term efficacy, and cost, to these therapeutic options for AMD becoming mainstream (94, 95).

## Author Contributions

All authors listed have made a substantial, direct, and intellectual contribution to the work and approved it for publication.

## Conflict of Interest

ML and GL are co-founders of Vision Engineering Italy srl. Vision Engineering Italy srl has no commercial or financial interest in the methods and products described herein. The remaining author declares that the research was conducted in the absence of any commercial or financial relationships that could be construed as a potential conflict of interest.

## Publisher's Note

All claims expressed in this article are solely those of the authors and do not necessarily represent those of their affiliated organizations, or those of the publisher, the editors and the reviewers. Any product that may be evaluated in this article, or claim that may be made by its manufacturer, is not guaranteed or endorsed by the publisher.

## References

[1] United Nations. World Population Ageing 2019. Highlights. United Nations (2019).

[2] WHO. Global Data on Visual Impairments 2010. Geneva: WHO (2012).

[3] Ferris FL Wilkinson CP Bird A Chakravarthy U Chew E Csaky K. Clinical classification of age-related macular degeneration. Ophthalmology. (2013) 120:844–51. 10.1016/j.ophtha.2012.10.036 23332590 PMC11551519

[4] Smith W Assink J Klein R Mitchell P Klaver CC Klein BE. Risk factors for age-related macular degeneration: Pooled findings from three continents. Ophthalmology. (2001) 108:697–704. 10.1016/S0161-6420(00)00580-7 11297486

[5] Owen CG Jarrar Z Wormald R Cook DG Fletcher AE Rudnicka AR. The estimated prevalence and incidence of late stage age related macular degeneration in the UK. Br J Ophthalmol. (2012) 96:752–6. 10.1136/bjophthalmol-2011-301109 22329913 PMC3329633

[6] NIH. National Plan for Eye and Vision Research. NIH Publication n. 04-4288.

[7] Moshfeghi AA Lanitis T Kropat G Kuznik A Gibson A Feng H. Social cost of blindness due to AMD and diabetic retinopathy in the United States in 2020. Ophthalmic Surg Lasers Imaging Retina. (2020) 51:S6–14. 10.3928/23258160-20200401-01 32348529

[8] Jeffery RCH Mukhtar SA Lopez D Preen DB McAllister IL Mackey DA. Incidence of newly registered blindness from age-related macular degeneration in australia over a 21-year period: 1996-2016. Asia Pac J Ophthalmol. (2021) 10:442–9. 34534144 10.1097/APO.0000000000000415

[9] Alan F Cruess AF Zlateva G Xu X Soubrane G Pauleikhoff D. Economic burden of bilateral neovascular age-related macular degeneration: multi-country observational study. Pharmacoeconomics. (2008) 26:57–73. 10.2165/00019053-200826010-00006 18088159

[10] Casten RJ Rovner BW Tasman W. Age-related macular degeneration and depression: a review of recent research. Curr Opin Ophthalmol. (2004) 15:181–3. 10.1097/01.icu.0000120710.35941.3f 15118503

[11] Ho L van Leeuwen R Witteman JC van Dujin CM Uitterlinden AG Hofman A. Reducing the genetic risk of age-related macular degeneration with dietary antioxidants, zinc, and omega-3 fatty acids: the Rotterdam study. Arch Ophthalmol. (2011) 129:758e766. 10.1001/archophthalmol.2011.141 21670343

[12] de Koning-Backus APM Buitendijk GHS Kiefte-de Jong JC Coljin JM Hofman A Vingerling JR. Intake of vegetables, fruit, and fish is beneficial for age-related macular degeneration. Am J Ophthalmol. (2019) 198:70–9. 10.1016/j.ajo.2018.09.036 30312575

[13] Merle BMJ Cougnard-Grégoire A Korobelnik J-F Schalch W Etheve S Rougier M-B. Plasma lutein, a nutritional biomarker for development of advanced age-related macular degeneration: the alienor study. Nutrients. (2021) 13:2047. 10.3390/nu13062047 34203817 PMC8232705

[14] Bernstein PS. Nutritional interventions against age-related macular degeneration. Acta Hortic. (2009) 841:103–12. 10.17660/ActaHortic.2009.841.10 20190863 PMC2826786

[15] Tan JS Wang JJ Flood V Rochtchina E Smith W Mitchell P. Dietary antioxidants and the long-term incidence of age-related macular degeneration: the Blue Mountains Eye Study. Ophthalmology. (2008) 115:334–41. 10.1016/j.ophtha.2007.03.083 17664009

[16] Moeller SM Parekh N Tinker L Ritenbaugh C Blodi B Wallace RB. Associations between intermediate age-related macular degeneration and lutein and zeaxanthin in the Carotenoids in Age-related Eye Disease Study (CAREDS): ancillary study of the Women's Health Initiative. Arch Ophthalmol. (2006) 124:1151–62. 10.1001/archopht.124.8.1151 16908818

[17] The Age-Related Eye Disease Study 2 Research Group. Lutein-zeaxanthin and omega-3 fatty acids for age-related macular degeneration. the age-related eye disease study 2 (AREDS2) randomized clinical trial. J Am Med Assoc. (2013) 309:2005–015. 10.1001/jama.2013.4997 23644932

[18] Lombardo M Serrao S Devaney N Parravano M Lombardo G. Adaptive optics technology for high-resolution retinal imaging. Sensors. (2013) 13:334–66. 10.3390/s130100334 23271600 PMC3574679

[19] Klein R Klein BE Knudtson MD Wong TY Cotch MF Liu K. Prevalence of age-related macular degeneration in 4 racial/ethnic groups in the multi-ethnic study of atherosclerosis. Ophthalmology. (2006) 113:373–80. 10.1016/j.ophtha.2005.12.013 16513455

[20] Colijn JM Buitendijk GHS Prokofyeva E Alves D Cachulo ML Khawaja AP. European Eye Epidemiology (E3) consortium. Prevalence of age-related macular degeneration in Europe: the past and the future. Ophthalmology. (2017) 2017:S0161–6420. 10.1016/j.ophtha.2017.05.035 28712657 PMC5755466

[21] Wong WL Su X Li X Cheung CM Klein R Cheng CY. Global prevalence of age-related macular degeneration and disease burden projection for 2020 and 2040: a systematic review and meta-analysis. Lancet Glob Health. (2014) 2:e106–116. 10.1016/S2214-109X(13)70145-1 25104651

[22] Rein DB Zhang P Wirth KE Lee PP Hoerger TJ McCall N. The economic burden of major adult visual disorders in the United States. Arch Ophthalmol. (2006) 124:1754–60. 10.1001/archopht.124.12.1754 17159036

[23] Rein DB Wittenborn JS Zhang X Honeycutt AA Lesesne SB Saaddine J. Forecasting age-related macular degeneration through the year 2050: the potential impact of new treatments. Arch Ophthalmol. (2009) 127:533–40. 10.1001/archophthalmol.2009.58 19365036

[24] Brown GC Brown MM Sharma S Stein JD Roth Z Campanella J. The burden of age-related macular degeneration: a value-based medicine analysis. Trans Am Ophthalmol Soc. (2005) 103:173–84. 17057801 PMC1447589

[25] Friedman DS O'Colmain BJ Munoz B Tomany SC McCarty C de Jong PTVM. Prevalence of age-related macular degeneration in the United States. Arch Ophthalmol. (2004) 122:564–72. 10.1001/archopht.122.4.564 15078675

[26] Chopdar A Chakravarthy U Verma D. Age related macular degeneration. BMJ. (2003) 326:485–8. 10.1136/bmj.326.7387.485 12609947 PMC1125371

[27] Maller JB Fagerness JA Reynolds RC Neale BM Daly MJ Seddon JM. Variation in complement factor 3 is associated with risk of age-related macular degeneration. Nat Genet. (2007) 39:1200–1. 10.1038/ng2131 17767156

[28] Sobrin L Ripke S Yu Y Fagerness J Bhangale TR Tan PL. Heritability and genome-wide association study to assess genetic differences between advanced Age-Related Macular Degeneration subtypes. Ophthalmology. (2012) 119:1874–85. 10.1016/j.ophtha.2012.03.014 22705344 PMC3899891

[29] Fritsche LG Igl W Bailey JN Grassman F Sengupta S Bragg-Gresham JL. A large genome-wide association study of age-related macular degeneration highlights contributions of rare and common variants. Nat Genet. (2016) 48:134e143. 10.1038/ng.3448 26691988 PMC4745342

[30] Wu J Sun X. Complement system and age-related macular degeneration: drugs and challenges. Drug Des Devel Ther. (2019) 13:2413e2425. 10.2147/DDDT.S206355 31409975 PMC6650090

[31] Colijn JM Meester-Smoor M Verzijden T de Breuk A Silva R Merle BMJ. Genetic risk, lifestyle, and age-related macular degeneration in europe: the EYE-RISK consortium. Ophthalmology. (2021) 128:1039–49. 10.1016/j.ophtha.2020.11.024 33253757

[32] Seddon JM Reynolds R Yu Y Daly MJ Rosner B. Risk models for progression to advanced age-related macular degeneration using demographic, environmental, genetic, and ocular factors. Ophthalmology. (2011) 118:2203–11. 10.1016/j.ophtha.2011.04.029 21959373 PMC4097877

[33] Chakravarthy U Wong TY Fletcher A Piault E Evans C Zlateva G. Clinical risk factors for age-related macular degeneration: a systematic review and meta-analysis. BMC Ophthalmol. (2010) 10:31. 10.1186/1471-2415-10-31 21144031 PMC3009619

[34] Loprinzi PD Swenor BK Ramulu PY. Age-related macular degeneration is associated with less physical activity among US adults: cross-sectional study. PLoS ONE. (2015) 10:e0125394. 10.1371/journal.pone.0125394 25933421 PMC4416755

[35] Carneiro Â José Paulo Andrade JP. Nutritional and lifestyle interventions for age-related macular degeneration: a review. Oxid Med Cell Longev. (2017) 2017:6469138. 10.1155/2017/6469138 28154734 PMC5244028

[36] Mares JA Voland RP Sondel SA. Healthy lifestyles related to subsequent prevalence of age-related macular degeneration. Arch Ophthalmol. (2011) 129:470–80. 10.1001/archophthalmol.2010.314 21149749 PMC3075357

[37] Koushan K Rusovici R Li W Ferguson LR Chalam KV. The role of lutein in eye-related disease. Nutrients. (2013) 5:1823–39. 10.3390/nu5051823 23698168 PMC3708350

[38] Qingning Bian Gao S Zhou J Qin J Taylor A Johnson EJ. Lutein and zeaxanthin supplementation reduces photo-oxidative damage and modulates the expression of inflammation-related genes in retinal pigment epithelial cells. Free Radic Biol Med. (2012) 53:1298–307. 10.1016/j.freeradbiomed.2012.06.024 22732187 PMC3744865

[39] Subczynski WK Wisniewska A Widomska J. Location of macular xanthophylls in the most vulnerable regions of photoreceptor outer-segment membranes. Arch Biochem Biophys. (2010) 504:61–6. 10.1016/j.abb.2010.05.015 20494651 PMC2957566

[40] Bhosale P Li B Sharifzadeh M Gellermann W Frederick JM Tsuchida K. Purification and partial characterization of a lutein-binding protein from human retina. Biochemistry. (2009) 48:4798–807. 10.1021/bi9004478 19402606

[41] Yemelyanov AY Katz NB Bernstein PS. Ligand-binding characterization of xanthophyll carotenoids to solubilized membrane proteins derived from human retina. Exp Eye Res. (2001) 72:381–92. 10.1006/exer.2000.0965 11273666

[42] Bone RA Landrum JT Fernandez L Tarsis SL. Analysis of the macular pigment by HPLC: retinal distribution and age study. Invest Ophthalmol Vis Sci. (1988) 29:843–9. 3372161

[43] Chen SF Chang Y Wu JC. The spatial distribution of macular pigment in humans. Curr Eye Res. (2001) 23:422–34. 10.1076/ceyr.23.6.422.6963 12045892

[44] Trieschmann M van Kuijk FJ Alexander R Hermans P Luthert P Bird AC. Macular pigment in the human retina: histological evaluation of localization and distribution. Eye. (2008) 22:132–7. 10.1038/sj.eye.6702780 17401321

[45] Widomska J Subczynski WK. Why has Nature chosen Lutein and Zeaxanthin to protect the retina? J Clin Exp Ophthalmol. (2014) 5:326. 10.4172/2155-9570.1000326 24883226 PMC4038937

[46] Rapp LM Maple SS Choi JH. Lutein and zeaxanthin concentrations in rod outer segment membranes from perifoveal and peripheral human retina. Invest Ophthalmol Vis Sci. (2000) 41:1200–9. 10752961

[47] Landrum JT Bone RA. Lutein, zeaxanthin, and the macular pigment. Arch Biochem Biophys. (2001) 385:28–40. 10.1006/abbi.2000.2171 11361022

[48] Ratnayake K Payton JL Meger ME Godage NH Gionfriddo E Karunarathne A. Blue light-triggered photochemistry and cytotoxicity of retinal. Cell Signal. (2020) 69:109547. 10.1016/j.cellsig.2020.109547 31982549 PMC7083221

[49] Kijlstra A Tian Y Kelly ER Berendschot TSJM. Lutein: more than just a filter for blue light. Prog Ret Eye Res. (2012) 31:303e315. 10.1016/j.preteyeres.2012.03.002 22465791

[50] Rodriguez-Carmona M Kvansakul J Harlow JA Köpcke W Schalch W Barbur JL. The effects of supplementation with lutein and/or zeaxanthin on human macular pigment density and colour vision. Ophthal Physiol Opt. (2006) 26:137–47. 10.1111/j.1475-1313.2006.00386.x 16460314

[51] Sasaki M Yuki K Kurihara T Miyake S Noda K Kobayashi S. Biological role of lutein in the light-induced retinal degeneration. J Nutr Biochem. (2012) 23:423–9. 10.1016/j.jnutbio.2011.01.006 21658930

[52] Liu R Wang T Zhang B Qin L Wu C Li Q. Lutein and zeaxanthin supplementation and association with visual function in age-related macular degeneration. Invest Ophthalmol Vis Sci. (2015) 56:252–8. 10.1167/iovs.14-15553 25515572

[53] Granado F Blázquez S Olmedilla B. Changes in catotenoid intake from fruit and vegetables in the spanish population over the period 1964 2004. Public Health Nutr. (2007) 10:1018–23. 10.1017/S1368980007662314 17381958

[54] Lucarini M Lanzi S D'Evoli L Aguzzi A Lombardi-Boccia G. Intake of vitamin A and carotenoids from the Italian population. Int J Vitam Nutr Res. (2006) 76:103–9. 10.1024/0300-9831.76.3.103 17048188

[55] Seddon JM Ajani UA Sperduto RD Hiller R Blair N Burton TC. Dietary carotenoids, vitamins A, C, and E, and advanced age-related macular degeneration. Eye disease case-control study group. J Am Med Assoc. (1994) 272:1413–20. 10.1001/jama.272.18.1413 7933422

[56] Seddon JM Ajani UA Sperduto RD Hiller R Blair N Burton TC. Dietary carotenoids, vitamins A, C, and E, and advanced age-related macular degeneration. Eye disease case-control study group. J Am Med Assoc. (1994) 272:1413–20. 10.1001/jama.1994.03520180037032 7933422

[57] Joachim N Mitchell P Burlutsky G Kifley A Wang JJ. The incidence and progression of age-related macular degeneration over 15 years: the blue mountains eye study. Ophthalmology. (2015) 122:2482–9. 10.1016/j.ophtha.2015.08.002 26383995

[58] Age-Related Eye Disease Study Research Group. The relationship of dietary carotenoids and vitamin A, E, and C intake with age-related macular degeneration in a case-control study: AREDS report no. 22. Arch Ophthalmol. (2007) 125:1225–123. 10.1001/archopht.125.9.1225 17846363

[59] Weigert G Kaya S Pemp B Sacu S Lasta M Werkmeister RM. Effects of lutein supplementation on macular pigment optical density and visual acuity in patient with age-related macular degeneration. Invest Ophthalmol Vis Sci. (2011) 52:8174–8. 10.1167/iovs.11-7522 21873668

[60] Berendschot TJM. Influence of lutein supplementation on macular pigment, assessed with two objective techniques. Invest Ophthalmol Vis Sci. (2000) 41:3322–6. 11006220

[61] Khachik F de Moura FF Chew EY Douglass LW Ferris FL Kim J. The effect of lutein and zeaxanthin supplementation on metabolites of these carotenoids in the serum of persons aged 60 or older. Invest Ophthalmol Vis Sci. (2006) 47:5234–42. 10.1167/iovs.06-0504 17122108

[62] The Age-Related Eye Disease Study 2 Research Group. The age-related eye disease study 2 (AREDS2): study design and baseline characteristics (AREDS2 report number 1). Ophthalmology. (2012) 119:2282–9. 10.1016/j.ophtha.2012.05.027 22840421 PMC3485447

[63] Musch DC. Evidence for including lutein and zeaxanthin in oral supplements for age-related macular degeneration. J Am Med Assoc. (2013) 2013:E1–3. 10.1001/jamaophthalmol.2013.7443 24309835

[64] Loane E McKay GJ Nolan JM Beatty S. Apolipoprotein E genotype is associated with macular pigment optical density. Invest Ophthalmol Vis Sci. (2010) 51:2636–43. 10.1167/iovs.09-4397 20107178

[65] Renzi LM Hammond BR Jr Dengler M Roberts R. The relation between serum lipids and lutein and zeaxanthin in the serum and retina: results from cross-sectional, case-control and case study designs. Lipid Health Dis. (2012) 11:33. 10.1186/1476-511X-11-33 22375926 PMC3310786

[66] Thomson LR Toyoda Y Langner A Delori FC Garnett KM Craft N. Elevated retinal zeaxanthin and prevention of light-induced photoreceptor cell death in quail. Invest Ophthalmol Vis Sci. (2002) 43:3538–49. 12407166

[67] Murray IJ Makridaki M van der Veen RL Carden D Parry NR Berendschot TT. Lutein supplementation over a 1 year period in early AMD might have a mild beneficial effect on visual acuity - the CLEAR study. Invest Ophthalmol Vis Sci. (2013) 54:1781–8. 10.1167/iovs.12-10715 23385792

[68] Eljarrat-Binstock E Domb AJ. Iontophoresis: a non-invasive ocular drug delivery. J Controlled Release. (2006) 110:479–89. 10.1016/j.jconrel.2005.09.049 16343678

[69] Myles ME Neumann DM Hill JM. Recent progress in ocular drug delivery for posterior segment disease: emphasis on transscleral iontophoresis. Adv Drug Delivery Rev. (2005) 57:2063–79. 10.1016/j.addr.2005.08.006 16310884

[70] Sousa-Martins D Sousa S Duarte J Marta M Lombardo M Lombardo G. Lutein reaches the retina following iontophoresis application. Invest Ophthalmol Vis Sci. (2016) 57:106.

[71] Lombardo M Villari V Micali N Roy P Sousa SH Lombardo G. Assessment of trans-scleral iontophoresis delivery of lutein to the human retina. J Biophotonics. (2018) 11:jbio.201700095. 10.1002/jbio.201700095 28700128

[72] Lombardo G Micali NL Villari V Serrao S Lombardo M. All-optical method to assess stromal concentration of riboflavin in conventional and accelerated UV-A irradiation of the human cornea. Invest Ophthalmol Vis Sci. (2016) 57:476–83. 10.1167/iovs.15-18651 26868750

[73] Hammond BR Wooten BR. Resonance Raman spectroscopic measurement of carotenoids in the skin and retina. J Biomed Opt. (2005) 10:054002. 10.1117/1.2116767 16292962

[74] Bernstein PS Yoshida MD Katz NB McClane RW Gellermann W. Raman detection of macular carotenoid pigments in intact human retina. Invest Ophthalmol Vis Sci. (1998) 39:2003–11. 9761278

[75] Age-Related Eye Disease Study Research Group. A simplified severity scale for age-related macular degeneration: AREDS Report No. 18. Arch Ophthalmol. (2005) 123:1570–4. 10.1001/archopht.123.11.1570 16286620 PMC1473206

[76] Damian I Nicoara SD. SD-OCT biomarkers and the current status of artificial intelligence in predicting progression from intermediate to advanced AMD. Life. (2022) 12:454. 10.3390/life12030454 35330205 PMC8950761

[77] Arrigo A Romano F Aragona E Di Nunzio C Battista M Bandello F. Optical coherence tomography angiography can categorize different subgroups of choroidal neovascularization secondary to age-related macular degeneration. Retina. (2020) 40:2263–9. 10.1097/IAE.0000000000002775 32032255

[78] Carroll J Kay D Scoles D Dubra A Lombardo M. Adaptive optics retinal imaging – clinical opportunities and challenges. Curr Eye Res. (2013) 38:709–21. 10.3109/02713683.2013.784792 23621343 PMC4031042

[79] Godara P Siebe C Rha J Michaelides M Carroll J. Assessing the photoreceptor mosaic over drusen using adaptive optics and SD-OCT. Ophthalmic Surg Lasers Imaging. (2010) 41(Suppl.): S104–8. 10.3928/15428877-20101031-07 21117594 PMC3124651

[80] Boretsky A Khan F Burnett G Hammer DX Ferguson RD van Kuijk F. *In vivo* imaging of photoreceptor disruption associated with age-related macular degeneration: a pilot study. Lasers Surg Med. (2012) 44:603–10. 10.1002/lsm.22070 22930575 PMC3593748

[81] Land ME Cooper RF Young J Berg E Kitchner T Xiang Q. Cone structure in subjects with known genetic relative risk for AMD. Optom Vis Sci. (2014) 91:939–49. 10.1097/OPX.0000000000000323 25014365 PMC4111779

[82] Gocho K Sarda V Falah S Sahel JA Sennlaub F Benchaboune M. Adaptive optics imaging of geographic atrophy. Invest Ophthalmol Vis Sci. (2013) 54:3673–80. 10.1167/iovs.12-10672 23620431

[83] Baraas RC Horjen Å Gilson SJ Pedersen HR. The relationship between perifoveal L-cone isolating visual acuity and cone photoreceptor spacing-understanding the transition between healthy aging and early AMD. Front Aging Neurosci. (2021) 13:732287. 10.3389/fnagi.2021.732287 34566629 PMC8458634

[84] Xu X Wang X Sadda SR Zhang Y. Subtype-differentiated impacts of subretinal drusenoid deposits on photoreceptors revealed by adaptive optics scanning laser ophthalmoscopy. Graefes Arch Clin Exp Ophthalmol. (2020) 258:1931–40. 10.1007/s00417-020-04774-w 32488329 PMC7442725

[85] Mujat M Ferguson RD Patel AH Iftimia N Lue N Hammer DX. High resolution multimodal clinical ophthalmic imaging system. Opt Express. (2010) 18:11607–21. 10.1364/OE.18.011607 20589021 PMC2958093

[86] Mariotti L Devaney N Lombardo G Lombardo M. Understanding the changes of cone reflectance in adaptive optics flood illumination retinal images over 3 years. Biomed Opt Expr. (2016) 7:2807–22. 10.1364/BOE.7.002807 27446708 PMC4948632

[87] Cooper RF Lombardo M Carroll J Sloan KR Lombardo G. Methods for investigating the local spatial anisotropy and the preferred orientation of cones in adaptive optics retinal images. Vis Neurosci. (2016) 33:e005. 10.1017/S0952523816000018 27484961 PMC5068353

[88] Werner JS Bieber ML Schefrin BE. Senescence of foveal and parafoveal cone sensitivities and their relations to macular pigment density. J Opt Soc Am A. (2000) 17:1918–32. 10.1364/JOSAA.17.001918 11059586 PMC2560986

[89] Gellermann W Ermakov IV Ermakova MR McClane RW Da-You Zhao Bernstein PS. *In vivo* resonant Raman measurement of macular carotenoid pigments in the young and the aging human retina. J Opt Soc Am A. (2002) 19:1172–86. 10.1364/JOSAA.19.001172 12049355

[90] Ermakov IV Sharifzadeh M Ermakova M Gellermann W. Resonance Raman detection of carotenoid antioxidants in living human tissue. J Biomed Opt. (2005) 10:064028. 10.1117/1.2139974 16409093 PMC3086339

[91] Bernstein PS Zhao DY Sharifzadeh M Ermakov IV Gellermann W. Resonance Raman measurement of macular carotenoids in the living human eye. Arch Biochem Biophys. (2004) 430:163–9. 10.1016/j.abb.2004.07.004 15369814

[92] Mohsen S Zhao DY Bernstein PS Gellermann W. Resonance Raman imaging of macular pigment distributions in the human retina. J Opt Soc Am A. (2008) 25:947–57. 10.1364/JOSAA.25.000947 18382494 PMC3079576

[93] Ermakov IV Ermakova MR Gellermann W. Simple Raman instrument for *in vivo* detection of macular pigments. Appl Spectrosc. (2005) 59:861–7. 10.1366/0003702054411616 16053555 PMC3079574

[94] Tan T-E Fenner BJ Barathi VA Tun SBB Wey YS Tsai ASH. Gene-based therapeutics for acquired retinal disease: opportunities and progress. Front Genet. (2021) 12:795010. 10.3389/fgene.2021.795010 34950193 PMC8688942

[95] Tan CS Ngo WK Chay IW Ting DS Sadda SR. Neovascular age-related macular degeneration (nAMD): a review of emerging treatment options. Clin Ophthalmol. (2022) 16:917–33. 10.2147/OPTH.S231913 35368240 PMC8965014

