# Supplementary material for: Challenges in Age-Related Macular Degeneration: From Risk Factors to Novel Diagnostics and Prevention Strategies
Source: Front Med (Lausanne). 2022 Jun 6;9:887104. doi: 10.3389/fmed.2022.887104 (PMC9207205; doi:10.3389/fmed.2022.887104)
Supplement: Supplementary file 1 [file Table_1.DOCX]

**Supplementary material**

**Two-photon microscopy analysis of scleral iontophoresis delivery of lutein in human eye globes**

The two-photon microscopy set-up was based on a Leica DM6000CS (Leica Microsystems GmbH, Germany) upright microscope configured to enable high sensitivity imaging. A Ti:sapphire laser (VISION II, Coherent, CA, USA) with an integrated proprietary prism-based unit was used as excitation source. This pulsed laser presents a tunable frequency range from 680 to 1080 nm, operating with a pulse width of 140 fs (measured at the sample plane) at 80 MHz of repetition rate. The laser power was attenuated by an Electro-Optical-Modulator (EOM) and then coupled into a Leica SP8-Spectral Scan-Head (Leica Microsystems GmbH, Germany) where it passed through the x-y scanning module, allowing the scanning in the x-y focal plane, before being focused by a Leica HCX IRAPO 25X/095 NA IRAPO, water immersion objective with a working distance of 2.5 mm corrected for multi-photon excitation and designed to have best imaging for deep tissue. Two-photon excitation at 835 nm was used and the two-photon emission fluorescence (TPEF) intensity signal was collected in backward direction by a non descanned detector (NDD1) for reflected light reflection. The light was first filtered by an IR filter SP680 and then encountered a dichroic beam splitter (Di02R405-25x36 Semrock Inc) and entered into the NDD1, in which the reflected light was filtered by a band pass filter 550/88 (FF01-550/88-25 Semrock Inc).

Before starting two-photon image acquisition on ocular tissues, several stacks on 0.005%, 0.002% and 0.001% diluted lutein formulation (in 0.9% sodium chloride) were acquired in order to correlate the fluorescence intensity emitted by the lutein formulation with the number of lutein liposome particles in the image plane. To this scope, an image processing routine was developed and used to count the particles present in the image plane. The number of lutein liposome particles decreased linearly (R^2^=0.994) with decreasing concentration of lutein from 0.005% to 0.001%. The average dimension of lutein liposome particles was ≥5 µm and was the same among the various diluted lutein formulation. Only-liposome lutein-free solution, which did not emit any TPEF signal, has been used as a negative control.

The eye globes were explanted between 3 and 19 hours after death (mean cadaver time: 12.7 ± 6.6 hours) and immediately preserved at 4° C in corneal storage medium enriched with 6% dextran. The mean donor age was 63.6 ± 5.9 years. The mean endothelial cell density was 2183 ± 41 cells/mm^2^. Each eye globe was mounted into a purpose-designed holder for TPEF imaging and connected, via tubing, to a column manometer, filled with 0.9% sodium chloride solution, in order to maintain the pressure inside the eye at 15 mmHg during experiment. In the eyes that underwent scleral iontophoresis, the passive electrode was placed in the optic nerve. The active electrode, consisting in a plastic bath tube, was applied to the scleral surface. In all cases, the plastic tube was filled with the liquid 0.1% lutein formulation. The current was set at 2.5 mA and delivered for 4 minutes. After iontophoresis, the eye globes were maintained, facing upward, in the eye holder with the pressure inside the eye at 15 mmHg for 1 hour. Thereafter, the retinal tissue was isolated without inducing gross damage that could compromise their use for high-resolution two-photon imaging. Dissection of retinal tissue and other ocular tissues was done using standardized protocol, which has been developed and validated by the Fondazione Banca degli Occhi del Veneto (<http://www.jove.com/video/3765/a-simplified-technique-for-situ-excision-cornea-evisceration-retinal>). The statistical power of the two-photon microscopy testing was calculated to reach 80% (β) to find differences of 6.0 arbitrary units (A.U.) in TPEF signal intensity (standard deviation: 20%; sample size: 6; allocation ratio: 0.5; α=0.05) between eyes that underwent scleral iontophoresis (n=4) and control eyes (n=2). The unpaired Student t-test was used to compare data between groups.

Images of the macula were collected by illuminating 500x500 µm areas, therefore covering the fovea and perifovea. The amount of lutein in controls and the relative increase of lutein delivered by scleral iontophoresis in the macula with respect to controls was calculated taking into account current knowledge on xanthophylls concentration distribution in the macula. In the normal human retina, the concentration of macular xanthophylls (ratio of lutein to zeaxanthin is 1:2) reaches a peak level ranging between 0.1 mM and 1 mM in the foveal center. This level decreases exponentially with increasing distances from the fovea, reaching low values at 300 µm from the fovea (i.e., 1.0 degree eccentricity). Accordingly, the maximum concentration of lutein in the macula is expected to range between 0.03 mM and 0.25 mM in the foveal area.

**Resonance Raman Spectroscopy of scleral iontophoresis delivery of lutein in human eye globes**

The resonant Raman spectroscopy (RRS) set-up included a 473 nm laser light source (0.75 mW/cm^2^ power at the retinal plane), which was directed through a dichroic filter and focused onto the sample with a 10-cm focal length lens. The backscattered light was imaged onto the entrance slit of a Raman spectrometer (ISA, Edison, NJ) passing through the dichroic filter, a focus lens and a notch filter. Raman signal intensity was recorded as counts per second (cps). The quantitative analysis of lutein from its typical resonance Raman peak (1530 cm^-1^) was performed by applying a minimization method in order to subtract the fluorescence background of the retinal compounds and a calibration procedure, as described in previous article.^77^ In this study, 1000 Raman counts corresponds to 1 ng/mm^2^ of xanthophyll pigment.

The eye globes were explanted between 3.5 and 12 hours after death (mean cadaver time: 7.2 ± 2.5 hours) and immediately preserved at 4° C in corneal storage medium enriched with 6% dextran. The mean donor age was 68 ± 3 years. The mean endothelial cell density was 2200 ± 20 cells/mm^2^.

Each eye globe was mounted into a purpose-designed holder and connected, via tubing, to a column manometer, filled with 0.9% sodium chloride solution, in order to maintain the pressure inside the eye at 15 mmHg during experiment. In the eyes that underwent scleral iontophoresis, the passive electrode was placed in the optic nerve. The active electrode, consisting in a plastic bath tube, was applied to the scleral surface. In all cases, the plastic tube was filled with the liquid 0.1% lutein formulation. The current was set at 2.5 mA and delivered for 4 minutes. After iontophoresis, the eye globes were maintained, facing upward, in the eye holder with the pressure inside the eye at 15 mmHg for 1 hour. Thereafter, the retinal tissue was isolated using standardized technique, as described above. The statistical power of the test was calculated (G*Power, available at http://www.gpower.hhu.de/) to reach 85% (β) to find average lutein concentration differences of 1.5 ng/mm^2^ (allocation ratio 3:1; standard deviation: 20%; α=0.05) between ocular tissues in treated and control eyes. The Wilcoxon test for unpaired data was used to compare data between groups.
